# The impact of informal caregiving on the mental health of health care workers during the COVID-19 pandemic—cross-sectional and longitudinal results from the VOICE study

**DOI:** 10.3389/fpubh.2025.1559518

**Published:** 2025-09-17

**Authors:** Gloria-Beatrice Wintermann, Anne Irmgard Bornhorn, Susann Steudte-Schmiedgen, Christian Albus, Andreas M. Baranowski, Petra Beschoner, Yesim Erim, Franziska Geiser, Lucia Jerg-Bretzke, Eva Morawa, Sabine Mogwitz, Kerstin Weidner

**Affiliations:** ^1^Department of Psychotherapy and Psychosomatic Medicine, Faculty of Medicine and University Hospital Carl Gustav Carus, TUD Dresden University of Technology, Dresden, Germany; ^2^Department of Psychosomatics and Psychotherapy, University of Cologne, Medical Faculty and University Hospital, Cologne, Germany; ^3^Department of Psychosomatic Medicine and Psychotherapy, University Hospital of Bonn, University Bonn, Bonn, Germany; ^4^Department of Psychosomatic Medicine and Psychotherapy, Ulm University Medical Center, Ulm, Germany; ^5^Department of Psychosomatic Medicine and Psychotherapy, University Hospital of Erlangen, Friedrich-Alexander University Erlangen-Nürnberg (FAU), Erlangen, Germany

**Keywords:** Severe Acute Respiratory Syndrome Coronavirus 2, Corona Virus Disease 2019 pandemic, healthcare workers, psychological distress, anxiety, depression, Patient Health Questionnaire, informal caregiver

## Abstract

**Background:**

The COVID-19 pandemic increased psychological distress among health care workers (HCWs). Those with informal caregiving responsibilities (ICs) may be especially vulnerable, but data remain limited.

**Methods:**

In a multicenter online survey conducted across four time points (T1–T4: 2020–2022) within the German healthcare system, HCWs with ICs were compared to those without (comparison group, CG). Psychological distress was assessed using validated measures of anxiety (Generalized Anxiety Disorder/GAD-2) and depression (Patient Health Questionnaire/PHQ-2). Group differences were analyzed using non-parametric Mann–Whitney U and chi-square tests. Longitudinal logistic regression analyses examined the impact of IC on psychological distress, controlling for sociodemographic, occupational, and psychological factors. A moderation analysis tested whether fear of infecting relatives influenced the salutogenic effect of optimism. All analyses were performed with multiply imputed data and a retrospective sample size justification was conducted.

**Results:**

ICs were more likely to be female, older, work in occupations other than medicine, work part-time, have children, and have more than 6 years of work experience. While ICs showed significantly higher PHQ-4 anxiety and depression than CG at T1 and T2, no significant differences could be shown for the other time points. IC emerged as a significant risk factor for increased psychological distress longitudinally, even after controlling for confounders. The protective effect of optimism was moderated by fear of infecting relatives at T1.

**Conclusion:**

HCWs with informal caregiving duties represent a vulnerable subgroup with increased psychological distress, especially during the early pandemic. This group may benefit from targeted support (e.g., flexible schedules, protective equipment, psychological interventions). Limitations include lack of pre-pandemic data and reliance on self-report. Findings underscore the importance of acknowledging and addressing overlapping care burdens in future health crises.

## Background

The Severe Acute Respiratory Syndrome (SARS) Coronavirus 2 (SARS-CoV-2) has been identified as the causative agent of a series of pneumonia cases that have occurred in clusters within the People’s Republic of China, with the first documented cases appearing in December 2019 (1), and in Germany, with the first cases appearing in January 2020 (2, 3). Within weeks, the virus spread, triggering an unprecedented global public health crisis unlike anything seen in the 21st century. As of January 2024, the Corona Virus Disease 2019 (COVID-19) pandemic has resulted in more than 770 million cases of infection and more than 7 million deaths worldwide (4).

In addition to the physical suffering caused by SARS-CoV-2, an increase in psychological distress has been observed, both in the general population and particularly among health care workers (HCWs) [for a systematic review: (5)]. During the COVID-19 pandemic, HCWs faced immense burdens due to increased workloads that exceeded already high baseline demands prior to the COVID-19 pandemic. For example, HCWs had to cope with COVID-related life-threatening illnesses with no curative treatment, additional ethically challenging triage situations, increased risk of personal infection, lack of protective equipment, and insufficient staff support, among others (6). A meta-analysis found that approximately one in five HCWs reported at least moderate levels of anxiety (7), while depression was mostly higher [e.g., (8, 9); systematic review: (5)] or equal (pooled prevalence of 22.8%) (10).

During the COVID-19 pandemic, restrictions on social contact, nationwide lockdowns and quarantines, the closure of care facilities and counseling centers, and the cancellation or postponement of medical procedures resulted in an increased care burden for employees with additional personal caregiving responsibilities (11, 12). Professional HCWs who provide unpaid practical care, usually to a disabled or seriously ill person in the community, most often a relative (13), are referred to as informal caregiving HCWs (hereafter referred to as ICs) or double-duty caregivers (14, 15).

Informal caregiving is expected to become increasingly important in the future, given the growing number of chronically ill people, older adults and the shortage of professional HCWs (16). Currently, between one in ten and one in five people in Europe provide informal care (17).

Based on previous research outside the COVID-19 pandemic (e.g., national caregiving studies in the United States), it is known that informal caregivers are predominantly middle-aged or older women and work at least part time (18, 19). They are at increased risk for psychological distress, especially when faced with limitations in their daily lives, such as coping with high levels of work–family conflict, financial strain due to part-time work, and lack of recreation during leisure time (20, 21). In line with this, a twofold increased risk of anxiety and depression has been reported among informal caregivers who experience personal limitations due to the demands of informal caregiving (20). However, beneficial effects have also been shown for specific forms of informal caregiving (e.g., companionship), particularly in terms of improved cognitive functioning in female caregivers (22), self-rated health (23), and perceived positive appraisal (24, 25).

During the COVID-19 pandemic across Europe, professional home care services were reduced due to staff shortages and/or epidemiological control measures (11). At the same time, informal caregivers were faced with acute exacerbations of their loved ones´ chronic conditions (26). In addition, they had to take on more responsibility for providing emotional support and managing the technology needed for telehealth and keeping in touch with loved ones. Based on the findings of the Global Carer Well-Being Index, a global research study on the health of informal/unpaid caregivers, the amount of time caregivers spend providing care increased significantly due to the pandemic [The Global Carer Well-Being Index, accessed May 25th, 2025 (27)] (28).

As a result, informal caregivers experienced higher levels of negative, stress-related emotions, such as fear and uncertainty, and reported lower levels of social support during the COVID-19 pandemic [for a qualitative systematic review: (13, 26)]. Approximately three-quarters of informal caregivers reported more burnout than ever before, while 61% reported a decline in their emotional and mental health during the COVID-19 pandemic [The Global Carer Well-Being Index, accessed on May 25th, 2025 (27)]. Since the outbreak of the COVID-19 pandemic, informal caregivers were more likely than non-caregivers to show mental distress (29, 30); e.g. feeling depressed or sad (19% vs. 16%) and anxious or nervous (26% vs. 21%) (11). This difference was particularly pronounced among parental caregivers (11) and with greater intensity of informal caregiving (31).

However, there is a lack of finding regarding the characterization of the group of informal caregiving HCWs (ICs). Above, results on the extent and course of psychological distress among them during the COVID-19 pandemic have not yet been published. It is also unknown whether informal caregiving is a distinct risk factor for increased anxiety/depression among HCWs during the COVID-19 pandemic, after controlling for already well-known sociodemographic (e.g., gender, age, living alone, having children), occupational (e.g., type of profession, working full-time/ part-time, work experience), pandemic-related (e.g., contact with COVID-19), and psychological risk factors (e.g., psychological distress at T1) (32–35).

HCWs may experience additional fears — such as fear of becoming infected themselves or transmitting the virus to others — and thus show increased levels of psychological distress (32, 34, 36–39) [for a systematic review: (5)]. The fear of infecting others (e.g., relatives) is thought to be more pronounced in HCWs with additional informal care responsibilities, thus potentially contributing to heightened psychological distress within this subgroup of double-duty caregivers. As a result, informal caregiving may be a distinct risk factor for increased anxiety/depression among HCWs during the COVID-19 pandemic.

Understanding specific risk and protective factors may help to initiate and justify the development of targeted intervention programs to help informal caregivers in general and informal caregiving HCWs in particular to maintain sufficient work capacity, mental health, and quality of life – both professionally and in their informal caregiving roles. In addition to examining informal caregiving and other known stressors, we focused on *optimism* as a psychological resource that may buffer against psychological distress. Optimism—conceptualized as a positive expectation about the future—has been associated with improved mental and physical health outcomes, including lower rates of depression and anxiety (40, 41). While often regarded as a dispositional trait, it can also be influenced through targeted interventions, making it a promising candidate for preventive strategies (42–44). Research among informal caregivers and HCWs has demonstrated that optimism contributes to emotional resilience, improved well-being, and more adaptive coping under pressure (24, 45). In the present study, we investigated whether higher levels of optimism were associated with lower psychological distress, and whether this association was influenced by fear of infecting vulnerable relatives—a concern especially pronounced in caregivers with dual responsibilities.

Drawing on the available literature, we propose the following hypotheses: (1) A higher proportion of middle-aged women working part-time is expected among the group of ICs compared to non-informally caregiving HCWs (CG, comparison group). (2) Given the increased demands of work and informal caregiving during the COVID-19 pandemic, we expect that ICs will report higher levels of psychological distress, defined as self-reported anxiety and depression (the primary outcome of the present study), than HCWs without informal caregiving responsibilities during all phases of the pandemic. Among ICs, a smaller increase in anxiety/ depression is anticipated, as they likely started from a higher baseline level at the onset of the pandemic. (3) We hypothesize that informal caregiving is an independent longitudinal risk factor for increased anxiety and depression, even after adjusting for sociodemographic (gender, age, living alone, children), work-related (occupation, part-time/full-time, work experience, contact with COVID-19), and psychological (anxiety/depression at onset of COVID-19 pandemic) covariates. (4) Secondary outcomes of interest included fear of becoming infected or infecting one’s family/relatives, and reduced recreation during leisure time, which are thought to contribute to higher psychological distress among ICs.

Furthermore, we assumed a salutogenic effect of optimism on psychological distress – particularly anxiety – and its potential moderation by the fear of infecting relatives.

## Methods

### Data collection

The present ongoing web-based survey entitled “VOICE” was conducted both cross-sectionally (with different cohorts) and longitudinally (with repeated measures in the same cohort) at the four time periods (T1, T2, T3, T4) during the COVID-19 pandemic in Germany. The primary objective was to assess the intensity, rate, and evolution of psychological distress, its risk factors, and protective resources in HCWs across four time periods: April 20th to July 5th, 2020 (T1), November 16th, 2020 to January 7th, 2021 (T2), May 26th to July 21st, 2021 (T3), February 7th to May 1st, 2022 (T4). For better understanding the context of the survey, we have shown the types of restrictions and pandemic measures that took place in Germany during the measurement periods in Supplementary Figure S1.

The VOICE study was conducted as a multi-wave cohort study within the framework of the egePan Unimed project (development, testing and implementation of regionally adaptive care structures and processes for evidence-based pandemic management). This project is part of the German Cooperation Network of University Medicine (NUM) and is supported by the German Federal Ministry of Education and Research (BMBF) (32).

The survey was developed by experienced professionals in psychosomatic medicine, psychology, and psychotherapy. It was piloted and revised several times to ensure clarity and feasibility. The psychosomatic departments of the university hospitals in Erlangen, Bonn, Ulm, Cologne, and Dresden disseminated the survey link to their staff via intranet or email, accompanied by at least one reminder. Additionally, the aforementioned entities disseminated the information in question to numerous municipal hospitals. Various professional networks also promoted participation in the survey (e.g., the Bavarian General Practitioners’ Association; the Federal Working Group of Social Pediatric Centers; the Federal Association of Psychosomatics and Medical Psychotherapy; the Federal Association of Occupational Medicine) and an Internet platform for physicians called Colliquio (also with at least one reminder).

The approximately 15 min survey was conducted in German using two academic online survey tools: Unipark (www.unipark.com, accessed April 1, 2021) and SoSci Survey (www.soscisurvey.de, accessed April 1, 2021). The initial survey consisted of 77 items (T1) and was slightly modified for subsequent waves. All versions of the questionnaires assessed experiences within the 2 weeks prior to completion.

Participants were asked to generate a personal code to identify multiple participation by the same individual and to enable longitudinal data linkage. Inclusion criteria were: a minimum age of 18 years, current employment in the German healthcare system, sufficient proficiency in the German language, and complete responses regarding caregiving situation (informal caregiving vs. no informal caregiving) and the primary outcome measure (PHQ-4). The exclusion criteria were aligned with the inclusion criteria mentioned above and included the following: age under 18 years, not being employed in the German healthcare system, incomplete data on the primary outcome (PHQ-4) or informal caregiving status, insufficient proficiency in the German language, and failure to provide informed consent to participate in the study. Respondents were categorized into four occupational groups: physicians, nurses, Medical Technical Assistants (MTAs, including medical, laboratory, radiology, or pharmaceutical technical assistants), and others (e.g., psychologists, chaplains).

Due to the heterogeneous recruitment strategy, it was not possible to measure the response rate for the overall sample. Since the present study originated from psychosomatic departments at five German university hospitals, most of the employees were recruited from these hospitals (T1: *n* = 3070/42.6%; T2: *n* = 4,197/ 65.8%; T3: *n* = 2548/73.6%; T4: *n* = 2590/63.5%). The remaining HCWs were recruited from other maximum-care hospitals, socio-pediatric centers, doctors’ offices, medical care centers, and other medical workplaces, e.g., occupational health services, nursing homes, outpatient care services, emergency services. For a detailed overview of the rate of participants from different medical workplaces (see Supplementary Table S1). Information on the response rates is provided by Morawa et al. (32).

### Data preparation

The survey was accessed a total of 23,287 times. Participants were excluded if they did not meet the inclusion criteria, failed to provide informed consent, completed only a trial version of the questionnaire, or did not complete all sociodemographic and occupational items relevant to this study. After data cleaning, a total of *N* = 21,129 healthcare workers (HCWs) were included in the final dataset and used for all subsequent analyses.

This dataset served as the basis for both cross-sectional and longitudinal analyses. Cross-sectional samples were formed separately for each time point (T1–T4), while longitudinal analyses focused on participants with complete data at both T1 and T2, as this subsample was the largest and corresponded to particularly critical phases of the COVID-19 pandemic.

Of the included participants, *n* = 7,215 provided complete data at T1 (April 2020), *n* = 6,375 at T2 (November 2020), *n* = 3,463 at T3 (May 2021), and *n* = 4,076 at T4 (February 2022). At each time point, participants were classified into either the informally caregiving group (IC) or the comparison group (CG) without caregiving responsibilities: T1: IC = 1,329, CG = 5,886; T2: IC = 1,049, CG = 5,326; T3: IC = 542, CG = 2,921; T4: IC = 554, CG = 2,959.

Detailed distributions of repeated participation and overlap across time points are presented in Supplementary Figures S2_1, S2_2.

### Measures

The measures used in the VOICE survey and relevant to the present study can be found in Supplementary material S3.

### Informal caregiving

Participants were asked whether they provided care for older adults, ill, or disabled individuals. No additional criteria beyond this core question were applied to determine classification as an informal caregiver. The exact wording of the item was: Participants were asked whether they provided care for older adults, ill, or disabled individuals (response options: yes, in my own household; yes, but not in my own household; no).

This operationalization is based on a single, binary self-report item and does not capture important dimensions such as caregiving duration, intensity, or subjective burden. These limitations reflect the constraints of the original study design, which prioritized feasibility in a large-scale, multi-wave survey context. Nevertheless, the classification aligns with common approaches in epidemiological caregiving research (13). We discuss the implications of this limited operationalization in the *Discussion* section.

### Symptoms of psychological distress

The primary outcome measure was symptoms of psychological distress (anxiety and depression) over the past 2 weeks using the Patient Health Questionnaire (PHQ)-4 (46). The PHQ-4 (see Supplementary material S3 for an overview of the relevant parts of the questionnaire used in the present study) is an ultra-brief screening tool consisting of four items, derived from the longer Patient Health Questionnaire-D (PHQ-D). It comprises two subscales: the PHQ-2 and the GAD-2. The PHQ-2 assesses depressive symptoms (e.g., anhedonia and depressed mood, as in “Felt down, depressed, or hopeless”), while the GAD-2 assesses generalized anxiety symptoms (e.g., “Feeling nervous, anxious, or on edge”).

Both subscales use a Likert-type scale ranging from 0 (“not at all”) to 3 (“nearly every day”), yielding subscale scores from 0 to 6. A cut-off of ≥3 on either subscale (GAD-2 or PHQ-2) and ≥6 on the total PHQ-4 score has been proposed to indicate clinically relevant symptoms. The psychometric properties of the PHQ-4 are well established (46, 47). In the present sample, the validated German version showed acceptable internal consistency, with Cronbach’s alpha values of 0.73 for the PHQ-2 and 0.74 for the GAD-2 (at T1). The PHQ-4 was administered at all four time points (T1, T2, T3, T4) during the COVID-19 pandemic.

### Sociodemographic factors

Several sociodemographic, work-related, and COVID-19-related variables were assessed as influential factors relevant to increased psychological distress in ICs. To collect sociodemographic data, respondents were asked to provide information about their gender (male, female or diverse), age (assessed using age groups only to ensure anonymity: 18–30 years, 31–40 years, 41–50 years, 51–60 years, >60 years), living alone (yes/no), having children (yes/no), Participants were asked whether they provided care for older adults, ill or disabled relatives (yes/no) (see Supplementary material S3).

### Working conditions during the COVID-19 pandemic

Working conditions during the COVID-19 pandemic were assessed based on the following variables: occupation (physician, nurse, Medical Technical Assistant assistance/MTA, others), years of work experience (<3 years, 3–6 years, >6 years), employment status (full-time/part-time), having contact with either COVID-19 infected patients proved by a test or contaminated material during work. The latter two categories were combined into a single variable: contact with COVID-19 (see Supplementary material S3).

### COVID-19 related working conditions

Other working conditions were assessed using five items rated on a scale from 1 (strongly disagree) to 5 (strongly agree). Of these, only the following item was relevant for the present analysis and included at both T1 and T2: “I can recover sufficiently during my free time” (see Supplementary material S3).

### Problems associated with the COVID-19 pandemic

Furthermore, potential stressors related to the COVID-19 pandemic were measured with nine items on a scale from 1 (strongly disagree) to 5 (strongly agree), referring to the previous 2 weeks. Of these, only the following two items were included in the present analysis, both at T1 and T2: “I was afraid of becoming infected” and “I was afraid of infecting relatives or my family.” In addition, a previous COVID-19 infection was assessed with a single item using a yes/no response format (see Supplementary material S3).

### Psychological resource: optimism

Participants were asked about their general optimism as an intra-individual, salutogenic and psychological variable. This was assessed at all time points using a single item: “How optimistic are you in general?,” rated on a 7-point Linkert scale ranging from 1 (not at all optimistic) to 7 (very optimistic) (see Supplementary material S3). For the present analyses, only optimism at T1 was of interest.

### Statistical analyses

All statistical analyses were conducted using SPSS version 29.0.0.0. Cross-sectional analyses were performed separately for each time point (T1–T4), while longitudinal analyses focused on participants with complete data at both T1 and T2. All analyses were based on the cleaned and preselected dataset (see Data Preparation section for details).

Participants who provided data at T1 but not at T2 or any subsequent time point (T3 or T4) were classified as dropouts. A dropout analysis was conducted to compare these individuals with those who continued participation (see Supplementary Table S2).

Descriptive variables are presented as frequencies and percentages. At all time points, ICs and CG were compared with respect to sociodemographic, work- and COVID-19-related variables using χ^2^-tests.

The primary outcomes (PHQ-2, GAD-2, PHQ-4) and secondary outcomes (fear of infection, sufficient recovery during leisure time) were tested for normality of distribution using the Kolmogorov–Smirnov test. As the data were not normally distributed, non-parametric Mann–Whitney U tests were used to compare group differences in outcome variables and additionally applied to assess gender differences. The effect size was calculated as the coefficient of determination using the formula *R*^2^ = z^2^/N (48). Interpretation can be based on Cohen’s recommendations, with cut-offs of *R*^2^ = 0.02/ 0.13/ 0.26 for small, medium, and large effects (49).

Frequencies and percentages of clinically relevant anxiety and depression were reported based on established cut-off scores (GAD-2/PHQ-2 ≥ 3; PHQ-4 ≥ 6). Chi-squared tests were used to compare ICs and the CG with respect to clinically relevant anxiety and depression rates and also with respect to the distinct trajectories of anxiety and depression symptoms (PHQ-4) from T1 to T2. These trajectories included persistence (PHQ-4 ≥ 6, both at T1 and T2), exacerbation (PHQ-4 < 6 at T1 and ≥ 6 at T2), resilience (PHQ-4 < 6, both at T1 and T2), and recovery (PHQ-4 ≥ 6 at T1 and < 6 at T2).

Additionally, the course of psychological distress (as a metric outcome) was analyzed longitudinally using general linear mixed models (GLMM) and maximum likelihood estimation. Time was considered a repeated-measures factor, and age group, gender, and COVID-19 exposure were considered between-subject factors.

To investigate longitudinal effects, binary logistic regression was used to assess whether informal caregiving at T1 predicted clinically relevant symptoms of anxiety and depression (PHQ-4 ≥ 6) at T2. This analysis controlled for sociodemographic, work-related, and COVD-19-related variables, as well as baseline PHQ-4 scores at T1. Similarly, the cross-sectional effects of informal caregiving on PHQ-4 at the same time point were analyzed while controlling for the aforementioned confounders. The latter were chosen based on the differences between the IC and the CG at T1, T2, T3, and T4, at a *p*-value of less than.05 (see Table 1; Supplementary Tables S4–S6). For the variable gender we also included the subgroup of gender-diverse participants. The type of workplace was included as an additional variable in separate logistic regression analyses. However, it was omitted from the final logistic regression models due to a lack of significance and parsimony. Odds ratios (OR) and 95% confidence intervals (CI) were reported.

**Table 1:** Descriptive statistical analyses, comparing the group of health care workers (HCWs) with informal caregiving (IC) and HCWs without informal caregiving (CG) at T1 (beginning of the COVID-19 pandemic), (between April to July 2020) (total *n* = 7,215).

	IC	CG	χ^2^ (*p*)
	*n* = 1,329 (18.4%)	*n* = 5,886 (81.6%)	
Gender, *n* (%)			
Female	1,078 (81.1)	4,422 (75.1)	
Male	246 (18.5)	1,453 (24.7)	
Divers	5 (0.4)	11 (0.2)	24.408 (<0.001)***
Age group in years, *n* (%)			
18–30	123 (9.3)	1,201 (20.4)	
31–40	159 (12.0)	1,459 (24.8)	
41–50	315 (23.7)	1,362 (23.1)	
51–60	595 (44.8)	1,479 (25.1)	
>60	137 (10.4)	385 (6.6)	320.529 (<0.001)***
Living alone, *n* (%)			
Yes	279 (21.0)	1,294 (22.0)	
No	1,050 (79.0)	4,592 (78.0)	0.625 (0.429)
Having children, *n* (%)			
Yes	855 (64.3)	3,194 (54.3)	
No	474 (35.7)	2,692 (45.7)	44.644 (<0.001)***
Occupation, *n* (%)			
Physician	284 (21.4)	1,551 (26.4)	
Nurse	242 (18.2)	1,008 (17.1)	
Medical-technical assistant	311 (23.4)	1,309 (22.2)	
Other^a^	492 (37.0)	2018 (34.3)	14.297 (0.003)**
Employment type, *n* (%)			
Full-time	771 (58.0)	3,707 (63.0)	
Part-time	558 (42.0)	2,179 (37.0)	11.358 (<0.001)***
Work experience, *n* (%)^5^			
<3 years	70 (5.3)	638 (10.8)	
3–6 years	87 (6.5)	624 (10.6)	
>6 years	924 (69.5)	3,694 (62.8)	
Not working in patient care	248 (18.7)	930 (15.8)	65.587 (<0.001)***
Direct contact with COVID-19, *n* (%)			
Yes	655 (49.3)	2,786 (47.3)	
No	674 (50.7)	3,100 (52.7)	1.657 (0.198)
COVID-19 infection, *n* (%)			
Yes	11 (0.8)	62 (1.1)	
No	1,318 (99.2)	5,824 (98.9)	0.551 (0.458)

a Other: dentist, paramedic, physiotherapist, psychologist, ergotherapist, speech therapist, pastor, student, scientist, midwife, IT and administration. CG, Comparison Group; IG, Informal Caregivers. ** *p* ≤ 0.01, *** *p* ≤ 0.001.

Finally, a moderation analysis [following Hayes’ PROCESS approach, (50)] was conducted to examine whether fear of infecting relatives or family at T1 moderated the association between general optimism at T1 and psychological distress (GAD-2, PHQ-2, PHQ-4) at T2.

We used Little’s MCAR test to analyze whether missing data on the primary outcome (PHQ-4) at all time points were missing completely at random (MCAR). The results indicated that the data were, in fact, not MCAR for the cross-sectional data (T1–T4). Next, we imputed the missing values using the Markov-Chain Monte Carlo (MCMC) algorithm, using a maximum of ten iterations and Predictive Mean Matching (PMM). The variables gender, having children, age group, occupation and employment type were defined as predictors, modelling the outcome variables (PHQ-4, PHQ-2, GAD-2). At T1, we imputed 852 (10.6%) missing data for the primary outcome PHQ-4, at T2 815 (11.3%), at T4 460 (10.1%). At measurement point T3, the dataset only included complete cases without missing data. This decision was made in accordance with the analytical strategy of the time, which prioritized the inclusion of complete data. However, this approach was later abandoned as it proved impractical for the other measurement points. Consequently, there are no missing values recorded for T3 because all incomplete cases were excluded from the dataset.

Regarding the secondary outcome parameters, 1,554 (19.3%)/2044 (28.4%)/167 (3.7%) missing data were imputed for “recovery during leisure time,” 497 (6.2%)/510 (7.1%)/881 (19.4%) “for fear of becoming infected” and “fear of infecting relatives,” at T1/T2/T4.

Missing data on the primary outcome (PHQ-4) at T1 or T2 (longitudinal data) were MCAR (*χ^2^* = 1.504, *df* = 2, *p* = 0.471). However, we assumed that the missing data were more likely to be missing at random than completely at random. Therefore, we also opted to use the MCMC algorithm to impute the missing values. A total of 1,146 (45.5%) PHQ-4/PHQ-2/GAD-2 values were replaced at T1 and 824 (32.7%) at T2.

To perform a sensitivity analysis, we repeated all analyses (where possible) with the imputed data to confirm the results of the complete-case analysis (see Supplementary Tables S7–S9, S11, S15).

All analyses were considered statistically significant at *p* ≤ 0.05. Where appropriate, Bonferroni–Holm corrections were applied to account for multiple comparisons.

### Power analysis

The VOICE study examined the psychological distress experienced by HCWs, regardless of their involvement in informal caregiving, during the pandemic. This study reanalyzed the data to focus on the impact of informal caregiving on HCWs´ psychological distress during the pandemic. Therefore, an *a priori* power analysis was not conducted. Instead, a posteriori power analysis was performed using G*Power (51). This analysis used a two-tailed Wilcoxon–Mann–Whitney U test to compare IC and CG. It assumed a small effect size of d = 0.1, an *α*-level of 0.05, statistical power of.80, and an allocation ratio of N2/N1 = 4. The analysis yielded a minimum total sample size requirement of 5,140 participants (IC: *n* = 1,028; CG: *n* = 4,112), which was met for T1 and T2 but not for T3 and T4. In contrast, the sample for the longitudinal logistic regression analysis (T1–T2) included a substantially smaller subsample (*N* = 965; IC: *n* = 153, CG: *n* = 812), which limits statistical power to detect small effects in longitudinal prediction models.

## Results

### Sociodemographic, work- and COVID-19-related characteristics

Table 1 presents descriptive statistics comparing ICs and the CG at time point T1 (the beginning of the COVID-19 pandemic, April to July 2020; total *n* = 7,215). Overall, 75.2% (*n* = 15,879) of all participating HCWs were female, 24.6% (*n* = 5,197) were male, and 0.3% (*n* = 53) identified as diverse.

At T1, 7215 HCWs completed the survey, including *n* = 1,329 in the IC group and *n* = 5,886 in the CG. Of the IC, 250 (18.8%) cared for an older adult, sick, or disabled person in their household, while 81.2% cared for someone outside their household. The groups differed significantly in terms of gender, with a higher proportion of women in the IC group (81.1%) compared to the CG group (75.1%). ICs were also older: 55.2% of the IC group were aged 51 years or older, compared to 31.7% in the CG.

In both groups, more than three-quarters lived with at least one other person. However, the ICs were more likely to have children (IC: 64.3%, CG: 54.3%) and to work as nurses, MTAs or in other healthcare professions (IC: 78.7%, CG: 73.7%), whereas a higher proportion of CG participants were physicians (IC: 21.4%, CG: 26.3%).

ICs were also more likely to work part-time (IC: 42.0%, CG: 37.0%) and had longer work experience, with 69.5% having more than 6 years compared to 62.8% in the CG. No significant group differences were found regarding occupational exposure to patients infected with COVID-19 or potentially infectious material. Likewise, no significant differences were observed in self-reported COVID-19 infection (IC: 0.8%, CG: 1.1%).

At the subsequent time points (T2 to T4), the group differences observed at T1 were largely maintained (see Supplementary Tables S4–S6). However, no significant differences were found in gender or employment status (part-time vs. full-time) at T3 and T4. At T2, ICs were more likely than CGs to report contact with COVID-19–infected patients or potentially infectious material (IC: 52.6%, CG: 47.3%). Most ICs cared for someone outside their own household, and this was comparable between time points (T2: 82.7%; T3: 79.3%; T4: 77.8%). The group comparisons were repeated using multiply imputed data (not shown), which confirmed the descriptive analyses from the complete-data analyses.

The results of the dropout analysis indicate that HCWs who were followed up at T2 were less likely to have children and less likely to have been infected with COVID-19 (Supplementary Table S2).

### Primary outcomes: depression and anxiety symptoms

**Figure 1:**
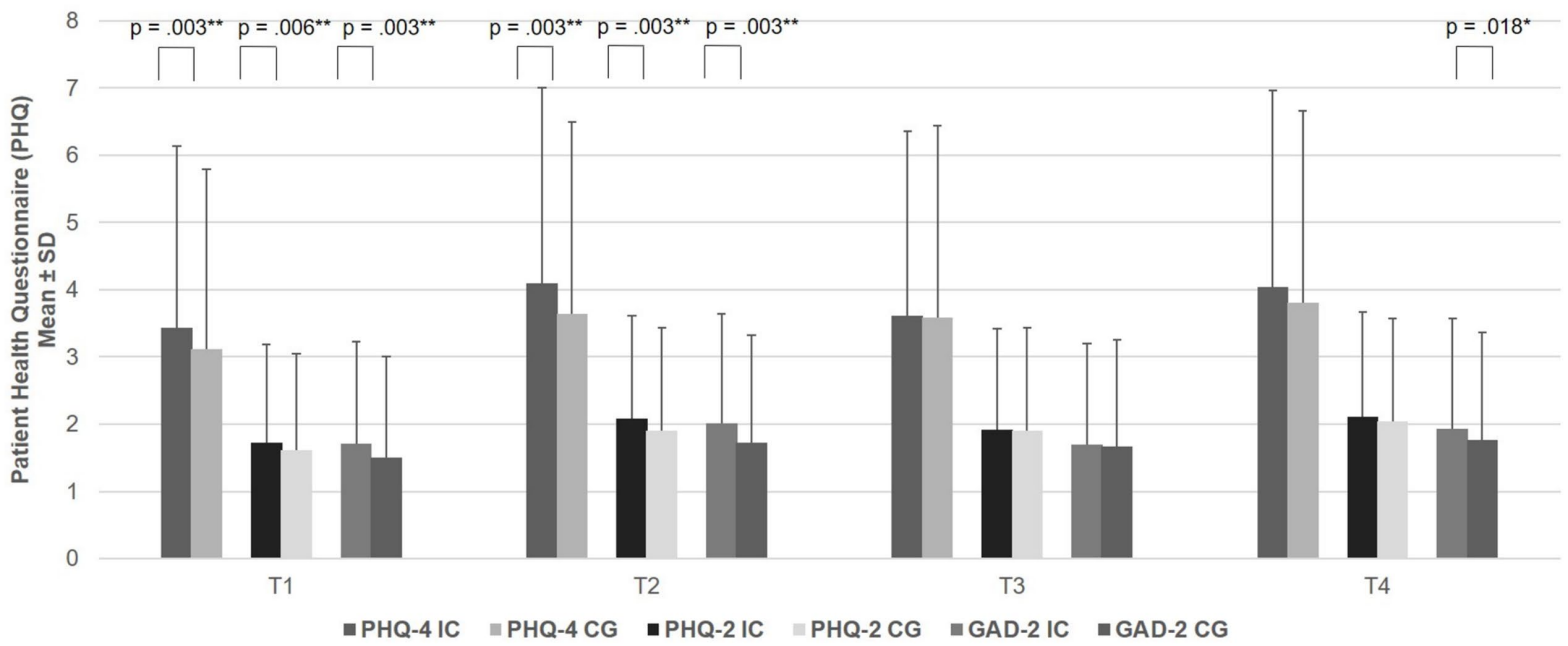
Comparison between anxiety (GAD-2), depression (PHQ-2) and anxiety and depression (PHQ-4), between health care workers with informal caregiving (IC) and the comparison group of health care workers without informal caregiving for family or relatives (T1, IC: 1329, CG: *n* = 5,886; T2, IC: *n* = 1,049, CG: *n* = 5,326; T3, IC: *n* = 542, CG: *n* = 2,921; T4, IC: *n* = 554, CG: *n* = 2,959). PHQ, Patient Health Questionnaire; GAD, Generalized Anxiety Disorder, * *p* ≤ 0.05, ** *p* ≤ 0.01.

Group differences in continuous scores for overall psychological distress (PHQ-4), depression (PHQ-2), and anxiety (GAD-2) were analyzed across all time points. PHQ-4 total scores indicated greater psychological distress among ICs at T1 and T2 (T1: *U* = 3618232.500, *p* < 0.001, Bonferroni-Holm corrected *p* = 0.003, *R*^2^ = 0.003; T2: *U* = 2516116.500, *p* < 0.001, Bonferroni-Holm corrected *p* = 0.003, *R*^2^ = 0.004), but not at T3 and T4. Similarly, PHQ-2 scores were also significantly higher among ICs at T1 and T2 (T1: *U* = 3728914.500, *p* = 0.006, Bonferroni-Holm corrected *p* = 0.006, *R*^2^ = 0.001; T2: *U* = 2608832.500, *p* < 0.001, Bonferroni-Holm corrected *p* = 0.003, *R*^2^ = 0.002), but not at T3 or T4. ICs reported significantly higher GAD-2 scores than the CG at T1, T2, and T4 (T1: *U* = 3580656.000, *p* < 0.001, Bonferroni-Holm corrected *p* = 0.003, *R*^2^ = 0.003; T2: *U* = 2487427.000, *p* < 0.001, Bonferroni-Holm corrected *p* = 0.003, *R*^2^ = 0.005; T4: *U* = 1052166.500, *p* = 0.006, Bonferroni-Holm corrected *p* = 0.018, *R*^2^ = 0.002) (see Figure 1; Table 2 for further information). The effect sizes can be considered small.

**Table 2:** Intensity of anxiety and depression (PHQ-4), depression (PHQ-2) and anxiety (GAD-2) at T1, T2, T3 and T4, cross-sectional analyses.

	IC, mean (SD)	CG, mean (SD)	U (*p*)	Coefficient of determination *R*^2^
*PHQ-4*				
T1^a^	3.43 (2.71)	3.12 (2.67)	3618232.500 (<0.001***) 0.003**#	0.003
T2^b^	4.09 (2.91)	3.64 (2.86)	2516116.500 (<0.001***) 0.003**#	0.004
T3^c^	3.62 (2.74)	3.58 (2.86)	771246.000 (0.338) 0.676#	0.000
T4^d^	4.04 (2.92)	3.80 (2.86)	1066521.500 (0.031*) 0.062#	0.001
*PHQ-2*				
T1^a^	1.72 (1.46)	1.61 (1.44)	3728914.500 (0.006**) 0.006**#	0.001
T2^b^	2.08 (1.54)	1.91 (1.52)	2608832.500 (<0.001***) 0.003**#	0.002
T3^c^	1.92 (1.50)	1.91 (1.53)	784626.000 (0.738) 0.738#	0.000
T4^d^	2.11 (1.56)	2.04 (1.53)	1101394.500 (0.363) 0.363#	0.000
*GAD-2*				
T1^a^	1.71 (1.52)	1.51 (1.50)	3580656.000 (<0.001***) 0.003**#	0.003
T2^b^	2.01 (1.63)	1.73 (1.60)	2487427.000 (<0.001***) 0.003**#	0.005
T3^c^	1.70 (1.50)	1.67 (1.59)	768603.000 (0.270) 0.810#	0.000
T4^d^	1.93 (1.64)	1.76 (1.60)	1052166.500 (0.006**) 0.018*#	0.002

a IC: *n* = 1,329, CG: *n* = 5,886.

b IC: *n* = 1,049, CG: *n* = 5,326.

c IC: *n* = 542, CG: *n* = 2,921.

d IC: *n* = 659, CG: *n* = 3,417.

* *p* ≤ 0.05; ** *p* ≤ 0.01; #Bonferroni-Holm-corrected *p*-values; CG, Comparison Group; GAD, Generalized Anxiety Disorder; IC, Informal Caregivers; PHQ, Patient Health Questionnaire.

In addition to mean scores, group differences in the proportion of participants exceeding clinical cut-offs were examined. Regarding clinically relevant anxiety and depression (PHQ-4 ≥ 6), ICs had significantly higher rates than the CG at T1 (20.0% vs. 16.4%; *χ^2^* = 9.742, contingency coefficient = 0.037, *p* = 0.002, Bonferroni-Holm corrected *p* = 0.008) and T2 (25.8% vs. 21.5%; *χ^2^* = 9.362, contingency coefficient = 0.038, *p* = 0.002, Bonferroni-Holm corrected *p* = 0.008), but not at T3 (*p* = 0.263, Bonferroni-Holm corrected *p* = 0.263) and T4 (*p* = 0.121, Bonferroni-Holm corrected *p* = 0.242).

For the PHQ-2, no significant group differences were observed at T1 (*χ^2^* = 2.307, contingency coefficient = 0.018, *p* = 0.129, Bonferroni-Holm corrected *p* = 0.387), T3 (*p* = 0.329, Bonferroni-Holm corrected *p* = 0.658), and T4 (*p* = 0.566, Bonferroni-Holm corrected *p* = 0.566), but a small yet significant difference was found at T2 that did not hold after Bonferroni-Holm correction (29.6% vs. 26.6%; *χ^2^* = 3.954, contingency coefficient = 0.025, *p* = 0.047, Bonferroni-Holm corrected *p* = 0.188).

Results for GAD-2 were similar to those for clinically relevant global psychological distress: significantly more ICs reached the clinical cut-off at T1 (23.6% vs. 19.8%; *χ^2^* = 9.871, contingency coefficient = 0.037, *p* = 0.002, Bonferroni-Holm corrected *p* = 0.006) and T2 (30.3% vs. 24.0%; *χ^2^* = 18.410, contingency coefficient = 0.054, *p* < 0.001, Bonferroni-Holm corrected *p* = 0.004), but not at T3 (*p* = 0.271, Bonferroni-Holm corrected *p* = 0.271) and T4 (*p* = 0.138, Bonferroni-Holm corrected *p* = 0.276).

The analyses were repeated using multiply imputed data and yielded the same results (see Supplementary Table S7). The results regarding clinically relevant symptoms of anxiety and depression using cut-offs were confirmed, except for PHQ-2 at T2. However, no group difference was found for PHQ-2 at T2 before Bonferroni-Holm correction (T1: PHQ-4 *χ^2^* = 8.299, contingency coefficient = 0.032, *p* = 0.004, Bonferroni-Holm corrected *p* = 0.008; PHQ-2 *χ^2^* = 2.648, contingency coefficient = 0.018, *p* = 0.104, Bonferroni-Holm corrected *p* = 0.104; GAD-2 *χ^2^* = 15.534, contingency coefficient = 0.044, *p* < 0.001, Bonferroni-Holm corrected *p* = 0.003; T2: PHQ-4 *χ^2^* = 5.656, contingency coefficient = 0.028, *p* = 0.017, Bonferroni-Holm corrected *p* = 0.034; PHQ-2 *χ^2^* = 2.285, contingency coefficient = 0.018, *p* = 0.131, Bonferroni-Holm corrected *p* = 0.131; GAD-2 *χ^2^* = 13.397, contingency coefficient = 0.043, *p* < 0.001, Bonferroni-Holm corrected *p* = 0.003; T4: *χ^2^* ≥ 0.331, contingency coefficient ≥ 0.009, *p* ≥ 0.125, Bonferroni-Holm corrected *p* ≥ 0.375).

### Effect of gender

To explore whether group differences between ICs and the CG were attributable to gender, separate Mann–Whitney U tests were conducted for men and women (see Supplementary Tables S8, S9).

At T1, no significant differences between ICs and the CG were observed among men for any of the primary outcomes (PHQ-4, PHQ-2, GAD-2; all Bonferroni-Holm corrected *p* ≥ 0.565, all *R*^2^ = 0.000). Among women, however, ICs reported significantly higher levels of psychological distress than the CG (PHQ-4: *U* = 2193312.000, *p* < 0.001, Bonferroni-Holm corrected *p* = 0.003, *R*^2^ = 0.003; PHQ-2: *U* = 2267471.000, *p* = 0.011, Bonferroni-Holm corrected *p* = 0.011, *R*^2^ = 0.001; GAD-2: *U* = 2163248.500, *p* < 0.001, Bonferroni-Holm corrected *p* = 0.003, *R*^2^ = 0.004). The effect sizes can be considered small.

At T2, significant differences were found in both men and women. Among men, ICs showed higher scores on PHQ-4 (*U* = 147374.500, *p* < 0.001, Bonferroni-Holm corrected *p* = 0.003, *R*^2^ = 0.009), PHQ-2 (*U* = 154100.000, *p* = 0.003, Bonferroni-Holm corrected *p* = 0.003, *R*^2^ = 0.005) and GAD-2 (*U* = 146790.500, *p* < 0.001, Bonferroni-Holm corrected *p* = 0.003, *R*^2^ = 0.010). Similarly, among women all primary outcomes were significantly elevated in ICs compared to the CG (PHQ-4: *U* = 1427590.000, *p* < 0.001, Bonferroni-Holm corrected *p* = 0.003, *R*^2^ = 0.003; PHQ-2: *U* = 1473451.000, *p* = 0.024, Bonferroni-Holm corrected *p* = 0.024, *R*^2^ = 0.001; GAD-2, *U* = 1409100.500, *p* < 0.001, Bonferroni-Holm corrected *p* = 0.003, *R*^2^ = 0.004). The effect sizes can be considered small.

No significant differences were found at T3 for either men (all Bonferroni-Holm corrected *p* ≥ 0.928, all *R*^2^ = 0.000) or women (all Bonferroni-Holm corrected *p* ≥ 0.719, all *R*^2^ = 0.000), as well as at T4 for men (all Bonferroni-Holm corrected *p* ≥ 0.468, 0.000 ≤ *R*^2^ ≤ 0.002). At T4, a significant difference was found only among women for GAD-2 (*U* = 622488.000, *p* = 0.034, *R*^2^ = 0.001), but this small effect did not remain significant after Bonferroni-Holm correction (Bonferroni-Holm corrected *p* = 0.102).

### Course of clinically relevant anxiety and depression (PHQ-4 ≥ 6) from T1 to T2

A greater proportion of ICs showed a persistent or worsening course of clinically relevant symptoms of anxiety and depression (PHQ-4 ≥ 6) between T1 to T2 compared to the CG. Specifically, 28.8% of ICs remained above or crossed the clinical threshold (persistent/worsening), compared to 21.5% of CGs, while 71.2% of ICs showed recovery or resilience, versus 78.5% of the CG (*χ^2^* = 3.909, contingency coefficient = 0.064, *p* = 0.048). However, this difference failed to reach statistical significance when multiply imputed data (*N* = 1,434) were used (*χ^2^* = 2.068, contingency coefficient = 0.038, *p* = 0.150). Descriptively, 22.4% of the CG experienced a persistent or worsening course of anxiety/depression, compared to 26.7% of the IC.

### The course of anxiety and depression from T1 to T4

A linear mixed model including all four time points (T1–T4) revealed a significant main effect of time on PHQ-4 scores [*F* (3, 731.98) = 4.720, *p* = 0.003], as well as a significant main effect of group [*F* (1, 1493.46) = 6.947, *p* = 0.008].

The fixed effect estimate indicated that PHQ-4 scores at T1 were significantly lower than at the subsequent time points (fixed parameter estimate = −1.415, *T* = −2.083, *p* = 0.038, 95% CI [−2.748, −0.081]). However, the fixed parameter estimate for group did not reach statistical significance (estimate = 0.543, *T* = 1.857, *p* = 0.064, 95% CI [−0.031, 1.117]). No significant interaction between group and time was found [*F* (3, 710.58) = 0.049, *p* = 0.986].

Regarding depression symptoms (PHQ-2), the linear mixed model revealed a significant main effect of time [*F* (3, 753.06) = 3.826, *p* = 0.010] and group [*F* (1, 1481.65) = 4.423, *p* = 0.036].

Although fixed effect estimates indicated lower PHQ-2 scores at T1 compared to later time points, the fixed effect estimate for T1 did not reach statistical significance (estimate = −0.652, *T* = −1.686, *p* = 0.092, 95% CI [−1.412, 0.107]). Similarly, the fixed effect estimate for group (IC vs. CG) was not significant (estimate = 0.126, *T* = 0.780, *p* = 0.436, 95% CI [−0.192, 0.444]). No significant interaction between group and time was observed [*F* (3, 730.24) = 0.191, *p* = 0.903].

For anxiety symptoms (GAD-2), the linear mixed model revealed a significant main effect of time [*F* (3, 760.47) = 3.694, *p* = 0.012] and group (IC vs. CG) [*F* (1, 1498.70) = 7.848, *p* = 0.005].

GAD-2 scores were significantly lower at T1 compared to T2 and T4, but not T3. The fixed effect estimate for T1 approached statistical significance (estimate = −0.710, *T* = −1.838, *p* = 0.067, 95% CI [−1.469, 0.049]). In contrast, the fixed effect estimate for group was significant, with higher overall anxiety scores in ICs compared to the CG (estimate = 0.419, *T* = 2.566, *p* = 0.010, 95% CI [0.098, 0.739]). No significant interaction between group and time was observed [*F* (3, 740.69) = 0.464, *p* = 0.708].

*Interaction time x gender:* Regarding the interaction between gender and time, the linear mixed model revealed no statistical significance for PHQ-4 or PHQ-2 (all *p* ≥ 302). However, it did reveal significance for GAD-2 [*F* (4, 1174.20) = 2.378, *p* = 0.050]. The fixed effect estimate indicated that GAD-2 scores at T2 significantly differed between men and women (fixed parameter estimate = 0.301, *T* = 2.663, *p* = 0.008, 95% CI [0.079, 0.522]). The values for women were significantly higher than those for men.

### Influence of informal caregiving on PHQ-4 scores (controlling for confounders)

Binary logistic regression analyses were conducted to assess the effect of informal caregiving on clinically relevant symptoms of depression and anxiety (PHQ-4 ≥ 6), controlling for relevant covariates.

At T1, informal caregiving was a significant predictor of elevated PHQ-4 scores (*OR* = 1.363, *p* < 0.001, 95% CI [1.147, 1.620]), after adjusting for gender, interaction gender x informal caregiving, age group, having children, living alone, occupation, work experience and employment type (Nagelkerkes *R*^2^ = 0.019, −2 Loglikelihood = 6513.214, *χ^2^* = 5.770, *df* = 8, *p* = 0.673). Both providing informal care in one’s own household (OR = 1.412, *p* = 0.040, 95% CI [1.015, 1.964]) and providing informal care outside one’s household (OR = 1.352, *p* = 0.001, 95% CI [1.124, 1.626]) were significantly associated with a higher PHQ-4 score.

Using multiply imputed data at T1 (*N* = 7,837), informal caregiving could be confirmed as a significant predictor (*OR* = 1.328, *p* < 0.001, 95% CI [1.131, 1.560], Nagelkerkes *R*^2^ = 0.019, −2 Loglikelihood = 7384.158, *χ^2^* = 8.351, *df* = 8, *p* = 0.400). Both providing care for an older adult, sick, or disabled person in one’s own household (OR = 1.509, *p* = 0.008, 95% CI [1.115, 2.042]) and outside one’s household (OR = 1.289, *p* = 0.004, 95% CI [1.084, 1.532]) were significant.

At T2, the effect of informal caregiving on the PHQ-4 remained significant (*OR* = 1.313, *p* = 0.003, 95% CI [1.094, 1.576]), controlling for the same variables as at T1 and adding the variable contact with COVID-19 (Nagelkerkes *R*^2^ = 0.028, −2 Loglikelihood = 6627.372, *χ^2^* = 7.339, *df* = 8, *p* = 0.500). Only providing informal care outside one’s household (OR = 1.302, *p* = 0.007, 95% CI [1.074, 1.580]) was significant. However, informal caregiving in one’s own household was associated with a similar, though not significant, increase in PHQ-4 scores (OR = 1.369, *p* = 0.085, 95% CI [0.985, 1.956]).

Using multiply imputed data at T2 (*N* = 6,957), informal caregiving could be confirmed as a significant predictor (*OR* = 1.285, *p* = 0.005, 95% CI [1.078, 1.532], Nagelkerkes *R*^2^ = 0.030, −2 Loglikelihood = 7231.154, *χ^2^* = 6.292, *df* = 8, *p* = 0.615). Only HCWs who cared for disabled individuals outside their own household showed a significantly higher PHQ-4 score (*OR* = 1.297, *p* = 0.006, 95% CI [1.077, 1.562]). However, informal caregiving in one’s own household was associated with a similar, though not significant, increase in PHQ-4 scores (*OR* = 1.230, *p* = 0.243, 95% CI [0.869, 1.741]).

At T3, no significant effect of informal caregiving on PHQ-4 was observed (OR = 1.049, *p* = 0.754, 95% CI [0.777, 1.418]), after controlling for relevant confounders (age, having children, occupation, work experience) (Nagelkerkes *R*^2^ = 0.062, −2 Loglikelihood = 2221.596, χ^2^ = 8.033, df = 8, *p* = 0.430).

At T4, informal caregiving continued to show a significant association with elevated PHQ-4 scores (*OR* = 1.467, *p* = 0.002, 95% CI [1.152, 1.869]), after controlling for age, having children, occupation, work experience and COVID-19 infection (Nagelkerkes *R*^2^ = 0.032, −2 Loglikelihood = 2923.430, *χ^2^* = 5.920, *df* = 8, *p* = 0.656).

Using multiply imputed data at T4 (*N* = 3,001), informal caregiving could be confirmed as a significant predictor (*OR* = 1.397, *p* = 0.005, 95% CI [1.104, 1.767], Nagelkerkes *R*^2^ = 0.031, −2 Loglikelihood = 3171.429, *χ^2^* = 2.610, *df* = 8, *p* = 0.956). Caring for a disabled individual in one’s own household had a greater impact (OR = 1.646, *p* = 0.028, 95% CI [1.057, 2.564]) than informal caregiving in another household (OR = 1.330, *p* = 0.033, 95% CI [1.023, 1.730]). However, both were significant.

In a longitudinal subsample including ICs and covariates measured at T1, informal caregiving was associated with a significantly increased likelihood of clinically relevant symptoms of psychological distress (PHQ-4) at T2 (Nagelkerkes *R*^2^ = 0.277, −2 Loglikelihood = 825.354, *χ^2^* = 8.250, *df* = 8, *p* = 0.409). Specifically, the odds of clinically relevant anxiety and depression is about 1.8 times greater in the group of ICs than in the CG (*OR* = 1.853, *p* = 0.016, 95% CI [1.120, 3.066]) (see Table 3). Caring for a disabled individual outside one’s own household had a small, though still significant, impact (OR = 1.784, *p* = 0.033, 95% CI [0.1.047, 3.041]) than informal caregiving in one’s own household (OR = 2.284, *p* = 0.125, 95% CI [0.795, 6.562]). The impact of informal caregiving remained consistent, even when the type of workplace was included as an additional covariate (*OR* = 1.857, *B* = 0.619, *SE* = 0.256, Wald = 5.828, *p* = 0.016, 95% CI [1.123, 3.070]).

**Table 3:** Influencing variables of clinically relevant anxiety and depression (PHQ-4 ≥ 6), at T2, longitudinal sample (data available at both T1 and T2) (*N* = 965, IC: *n* = 153, CG: *n* = 812).

	Regression coefficient B	SE	Wald	*P*	OR (95%CI)
Informal caregiving					
Yes	0.617	0.257	5.764	0.016*	1.853 (1.120, 3.066)
Gender					
Female	0.070	0.250	0.078	0.779	1.073 (0.657, 1.750)
*Gender x Informal Caregiving*	−0.583	0.707	0.680	0.410	0.558 (0.140, 2.231)
Age (years)					
31–40	−0.138	0.301	0.211	0.646	0.871 (0.483, 1.571)
41–50	−0.571	0.346	2.719	0.099	0.565 (0.287, 1.114)
51–60	−0.801	0.341	5.535	019*	0.449 (0.230, 0.875)
>60	−0.278	0.503	0.306	0.580	0.757 (0.283, 2.028)
Living alone					
Yes	−0.003	0.197	0.868	0.352	0.833 (0.566, 1.224)
Children					
Yes	0.109	0.228	0.229	0.632	1.116 (0.713, 1.745)
Occupation					
Nursing	0.312	0.273	1.308	0.253	1.367 (0.800, 2.334)
MTA	0.530	0.303	3.072	0.080	1.699 (0.939, 3.074)
Other	0.000	0.253	0.000	1.000	1.000 (0.609, 1.641)
Full-time					
Yes	0.221	0.200	1.220	0.269	1.248 (0.843, 1.848)
Work experience (years)					
3–6	−0.125	0.363	0.119	0.730	0.882 (0.433, 1.796)
>6	−0.356	0.350	1.036	0.309	0.700 (0.353, 1.391)
Not working with patients	0.204	0.376	0.295	0.587	1.226 (0.587, 2.560)
Contact with COVID-19					
Yes	0.202	0.187	1.169	0.280	1.223 (0.849, 1.763)
PHQ-4 ≥ 6 at T1	2.436	0.214	129.629	<0.001***	11.431 (7.515, 17.386)

Nagelkerkes *R*^2^ = 0.277, −2 Loglikelihood = 825.354, χ^2^ = 8.250, df = 8, *p* = 0.409.

CG, Comparison Group; GAD, Generalized Anxiety Disorder; IG, Informal Caregivers; MTA, medical technical assistant; PHQ, Patient Health Questionnaire; Because only one participant of a diverse gender was included in the longitudinal analyses (*n* = 1), we decided not to report the odds ratio for this subgroup.

The significant impact of informal caregiving was confirmed when multiply imputed data were used (Supplementary Table S10). Providing care outside one’s own household had a smaller, though still significant, impact (OR = 1.545, *p* = 0.041, 95% CI [1.018, 2.345]) than providing care in one’s own household (OR = 1.978, *p* = 0.069, 95% CI [0.947, 4.129]). The significant effect of informal caregiving remained stable when the type of workplace was included as an additional covariate (*OR* = 1.621, *B* = 0.483, *SE* = 0.197, Wald = 5.992, *p* = 0.014, 95% CI [1.101, 2.387]).

### Secondary outcomes

Secondary outcomes included fear of becoming infected, fear of infecting relatives or family members, and recovery during leisure time.

Among ICs, the fear of becoming infected was significantly higher than in the CG, at T1, T2, and T4. The fear of infecting others was significantly higher in ICs across all time points (T1–T4). In addition, ICs reported significantly less recovery during leisure time at T1, T2 and T4 (Table 4). Using multiply imputed data, significant differences were confirmed. However, ICs only showed a significantly increased fear of becoming infected at T1 and T2 (Supplementary Table S11). The effect sizes can be considered small.

**Table 4:** The fear of becoming infected, fear of infecting relatives/family and recovery during leisure time (each rated on a 5-point Likert Scale ranging between 1 “strongly disagree” to 5 “strongly agree”), were compared between informal caregivers (ICs) and the comparison group of health care workers without informal caregiving (CG).

	IC, mean (SD)	CG, mean (SD)	U (p)	Coefficient of determination *R*^2^
Fear of becoming infected				
T1^a^	2.88 (1.27)	2.61 (1.22)	3765295.000 (<0.001***) 0.003**#	0.007
T2^b^	3.21 (1.24)	3.05 (1.26)	2846092.500 (<0.001***) 0.003**#	0.002
T3^c^	2.24 (1.17)	2.12 (1.12)	747583.500 (0.031*) 0.062#	0.001
T4^d^	2.96 (1.34)	2.83 (1.36)	832230.000 (0.038*) 0.038*#	0.001
Fear of infecting relatives or family				
T1^a^	3.65 (1.27)	3.19 (1.35)	3458234.500 (<0.001***) 0.003**#	0.018
T2^b^	3.87 (1.19)	3.53 (1.30)	2608632.000 (<0.001***) 0.003**#	0.010
T3^e^	2.83 (1.41)	2.59 (1.35)	711333.000 (<0.001***) 0.003**#	0.004
T4^d^	3.60 (1.34)	3.19 (1.39)	728539.500 (<0.001***) 0.003**#	0.012
Recovery during leisure time				
T1^f^	2.96 (1.27)	3.08 (1.28)	2967992.500 (0.003**) 0.003**#	0.001
T2^g^	2.47 (1.17)	2.73 (1.21)	1570590.000 (<0.001***) 0.003**#	0.007
T3^c^	2.75 (1.23)	2.81 (1.22)	767863.500 (0.254) 0.254#	0.000
T4^h^	2.61 (1.15)	2.76 (1.18)	1191657.500 (0.002**) 0.004**#	0.002

a IC: *n* = 1,389, CG: *n* = 6,181.

b IC: *n* = 1,097, CG: *n* = 5,583.

c IC: *n* = 542, CG: *n* = 2,921.

d IC: *n* = 570, CG: *n* = 3,085.

e IC: *n* = 539, CG: *n* = 2,911.

f IC: *n* = 1,175, CG: *n* = 5,338.

g IC: *n* = 833, CG: *n* = 4,313.

h IC: *n* = 699, CG: *n* = 3,670.

** *p* ≤ 0.01; *** *p* ≤ 0.001; #Bonferroni-Holm-corrected *p*-values; CG, Comparison Group; GAD, Generalized Anxiety Disorder; IG, Informal Caregivers; PHQ, Patient Health Questionnaire.

### Moderation analysis: fear of infecting relatives or family as moderator between optimism at T1 and anxiety and depression (PHQ-4) at T2

A moderation analysis was conducted within the group of ICs (*n* = 152) to examine whether the fear of infecting relatives or family members at T1 moderated the association between general optimism at T1 and psychological distress at T2.

Results revealed a significant moderating effect of fear of infecting family or relatives at T1 on the relationship between optimism and GAD-2 anxiety (*β* = 0.196, *t* = 2.706, *p* = 0.008, 95% CI [0.053, 0.339], *R*^2^ = 0.161, *F* (3,148) = 9.451, *p* < 0.001), as well as PHQ-4 (*β* = 0.341, *t* = 2.575, *p* = 0.011, 95% CI [0.079, 0.602], *R*^2^ = 0.177, F (3,148) = 10.608, *p* < 0.001), but not on PHQ-2 (*β* = 0.145, *t* = 1.963, *p* = 0.052, 95% CI [−0.001, 0.291], *R*^2^ = 0.146, F (3,148) = 8.443, *p* < 0.001) (see Table 5).

**Table 5:** In an additional moderation analysis, the salutogenic effect of optimism at T1 on anxiety and depression (PHQ-4), depression (PHQ-2), and anxiety (GAD-2) at T2, moderated by the fear of infecting family or relatives at T1, was analyzed in the group of informal caregivers only (IC, *n* = 152).

Regressors	Outcome	Beta (95% CI)	*t*	*p*
General optimism at T1	GAD-2	−0.236 (−0.407, −0.064)	−2.714	0.007**
	PHQ-2	−0.340 (−0.516, −0.165)	−3.836	<0.001***
	PHQ-4	−0.576 (−0.890, −0.262)	−3.626	<0.001***
Fear of infecting relatives at T1 (moderator)	GAD-2	0.375 (0.175, 0.574)	3.707	<0.001***
	PHQ-2	0.252 (0.048, 0.456)	2.445	0.016*
	PHQ-4	0.627 (0.262, 0.992)	3.392	<0.001***
Interaction fear x general optimism	GAD-2	0.196 (0.053, 0.339)	2.706	0.008**
	PHQ-2	0.145 (−0.001, 0.291)	1.963	0.052
	PHQ-4	0.341 (0.079, 0.602)	2.575	0.011*

* *p* ≤ 0.05, ** *p* ≤ 0.01, *** *p* ≤ 0.001; CI, confidence interval.

**Figure 2:**
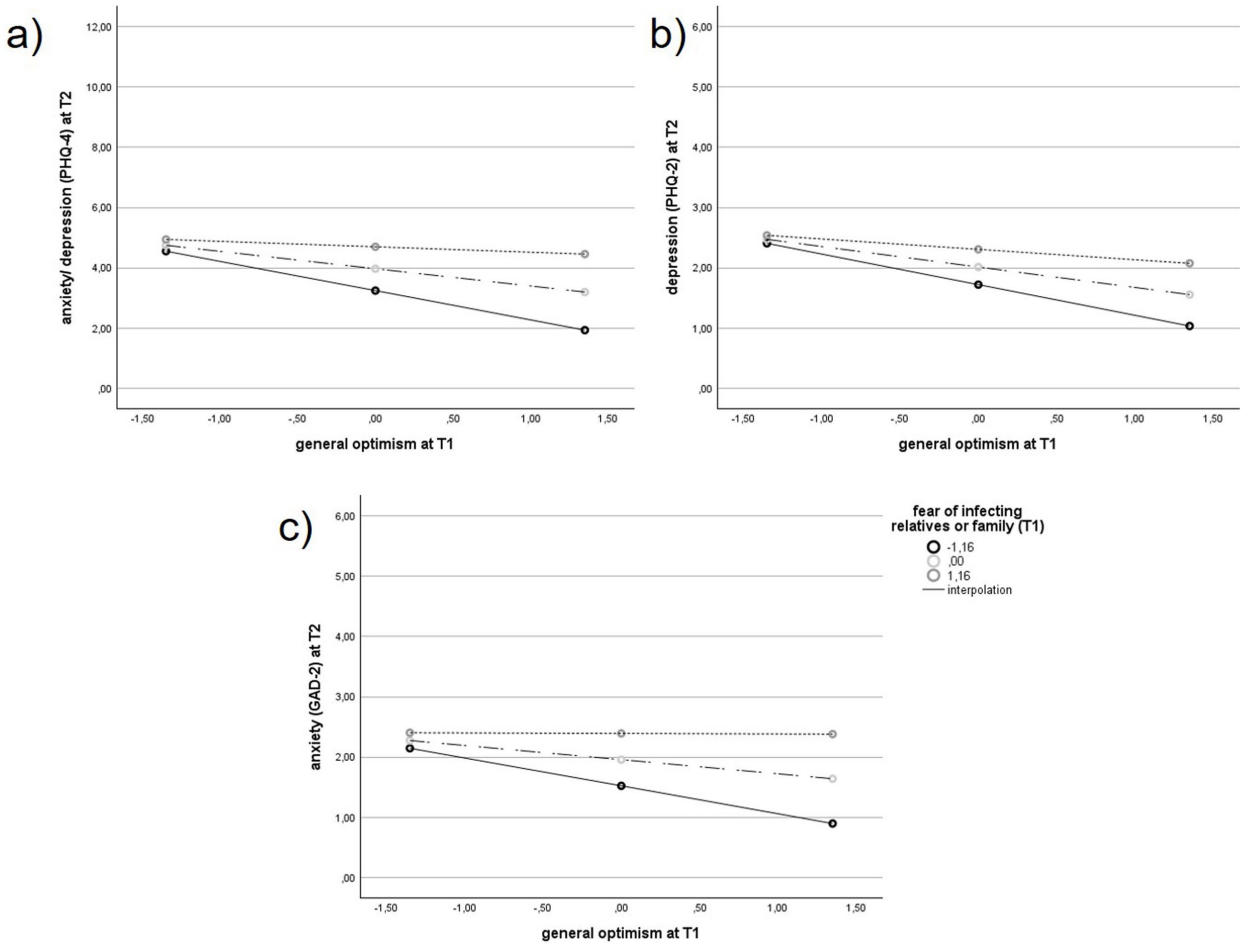
Correlation between general optimism at T1 and anxiety and depression (PHQ-4) **(a)**, depression (PHQ-2) **(b)**, anxiety (GAD-2) **(c)** at T2, depending from the intensity of the fear of infecting relatives or family in the group of health care workers (HCWs) with additional informal caregiving, *n* = 152. PHQ, Patient Health Questionnaire; GAD, Generalized Anxiety Disorder.

The protective effect of optimism was only present among ICs with low fear of infecting others. The effect, however, was absent under high fear of infecting others (see Figure 2). Simple slopes and Johnson-Neyman significance regions for PHQ-4, PHQ-2 and GAD-2 as outcomes are presented in the Supplementary Tables S12A,B–S14A,B.

The analyses were repeated using multiply imputed data for the primary outcomes and confirmed the results of wthe complete-case analyses. The fear of infecting family or relatives had a moderating effect on the relationship between optimism and GAD-2 anxiety at T1 (*β* = 0.133, *t* = 2.034, *p* = 0.043, 95% CI [0.004, 0.262], *R*^2^ = 0.100, *F* (3,220) = 8.151, *p* < 0.001). This effect was also observed for PHQ-4 (*β* = 0.304, *t* = 2.386, *p* = 0.018, 95% CI [0.053, 0.555], *R*^2^ = 0.086, *F* (3,220) = 6.854, *p* < 0.001). However, there was no such effect on PHQ-2 (*β* = 0.056, *t* = 0.811, *p* = 0.418, 95% CI [−0.079, 0.190], *R*^2^ = 0.057, F (3,220) = 4.394, *p* = 0.005) (Supplementary Table S15).

## Discussion

This present study examined psychological distress among HCWs with additional informal caregiving responsibilities (=ICs) during the COVID-19 pandemic. To our knowledge, this is the first study to focus specifically on a sample of HCWs in Germany who provided informal care to relatives in addition to their professional duties during the COVID-19 pandemic. Our primary objective was to investigate whether the group of ICs differed from HCWs without additional informal caregiving responsibilities – defined as comparison group (CG) – in terms of anxiety and depression.

### Depression and anxiety symptoms in informal caregiving HCWs (ICs)

The results suggest that ICs reported higher levels of anxiety and depression compared to the CG at the beginning of the pandemic (April to July 2020) and again at T2 (November 2020 to January 2021), but not at T3 or T4. Overall psychological distress — measured by the PHQ-4 (combined anxiety and depression score) and anxiety specifically by the GAD-2 (using metric scores for both)—was significantly higher among ICs than in the CG, at least during the early phase and throughout the first year of the pandemic.

Previous findings comparing informal caregivers and non-caregivers have already been published and demonstrated increased rates of psychological distress, e.g., anxiety, depression, among informal caregivers during the COVID-19 pandemic (11, 29–31, 52). Our finding is in contrast to a large Canadian longitudinal study, showing more frequent depressive but not anxiety symptoms in informal caregivers between April and December 2020 (53). However, no previous studies have focused specifically on HCWs who also provide informal care to relatives.

Interestingly, mean scores for clinically relevant levels of anxiety and depression (PHQ-4) in both the ICs and the CG were lower than those found in a normative German sample assessed during the first and second waves of the pandemic (47), as well as in a German general population sample surveyed at the onset of COVID-19 (54). This discrepancy may be explained by the fact that the participants in our present study were HCWs, a group known for their high levels of competence and resilience in managing challenging, potentially life-threatening situations (32).

### The impact of gender

The observed differences in psychological distress between ICs and the CG may partly reflect gender-related effects, as women were overrepresented in the IC group. Prior research has consistently identified female gender as a risk factor for higher levels of anxiety and depression, both in the general population and among HCWs during the pandemic (32–34, 55–58). Moreover, informal caregiving tends to have a greater negative impact on self-rated health and psychological burden in women than in men (59).

To explore these effects, we compared anxiety and depression levels between ICs and CG separately for men and women. At the beginning of the pandemic (T1), women in the IC group showed significantly higher anxiety and depression scores (PHQ-4, PHQ-2, GAD-2) than women in the CG. As the pandemic progressed (T2), psychological distress increased across genders, with both male and female ICs reporting significantly elevated symptoms compared to their respective CGs. These findings suggest that while gender may partly explain the initial group differences, informal caregiving itself emerged as an independent risk factor (at T1, T2, and T4), persisting even after controlling for gender and age in our longitudinal regression model.

Notably, male ICs also showed increased distress during T2, potentially reflecting a disruption of traditional caregiving roles and limited access to coping resources. Previous studies have highlighted that men often face higher barriers to seeking support and report lower social connectedness (53). In combination with restrictive public health measures (e.g., school closures, reduced social contact), this may have contributed to heightened psychological strain during this period. Taken together, gender can be considered both as a background variable and as a potential moderator of caregiving-related stress.

### The impact of fear of infection, optimism and recovery during leisure time on psychological distress

Fear of infecting family members emerged as a key contributor to psychological distress, particularly in informal caregiving HCWs. Due to their dual roles—exposure to COVID-19 at work and close contact with vulnerable relatives—ICs reported significantly higher fear of transmission at all time points. This concern was amplified by pandemic-related reductions in formal care services, increasing direct caregiving responsibilities and perceived infection risk. Consistent with previous findings, fear of infecting others was significantly associated with anxiety and depression and has been identified as a key predictor of mental health outcomes in HCWs during the pandemic (5, 28).

Psychological resources such as optimism may buffer these effects. In our study, optimism was associated with lower distress levels. This result aligns with previous work identifying optimism as a stronger predictor of mental health than demographic or occupational variables in HCWs (35). Optimism may facilitate adaptive coping strategies such as cognitive reappraisal and tolerance for uncertainty (60), and has been shown to support well-being in caregivers (24). In the present study, the impact of optimism was modified by the level of fear of infecting relatives, but only in the IC group. Specifically, the protective effect of optimism was only evident among ICs who had low levels of fear about infecting others. This finding highlights the role of situational stress in influencing optimism and confirms the state component of optimism.

However, optimism is not universally protective. High levels of optimism may also reduce perceived threat and lead to complacency, diminishing adherence to protective measures and unintentionally increasing the risk of virus transmission at home. This duality underscores the need for balanced interventions: psychological support programs should enhance positive coping (e.g., optimism, resilience, coherence), while also reinforcing risk awareness. Educational efforts, clear communication, and access to protective equipment remain essential, particularly for ICs who face elevated fear and responsibility. Future studies should explore the nuanced interaction between protective traits and situational stressors to inform targeted interventions [for a meta-analysis: (44)] (61).

Beyond fear of infection, lack of recovery during leisure time was a further predictor of increased psychological distress. ICs consistently reported lower recovery (except at T3) compared to the CG, and this factor showed even stronger associations with distress than fear of infection (data not shown). In addition to being more likely to be female, ICs were more likely to be middle-aged and to have multiple roles, such as having children. This has previously been shown in a census sample of informal caregivers in the United Kingdom (62), and it is suggested that reduced leisure time, and therefore recovery during leisure time, may also have contributed to increased psychological distress (32). Limited opportunities to rest and detach from responsibilities likely reflect the cumulative burden of multiple roles, such as caregiving, childcare, and professional duties. Targeted support measures, such as flexible work schedules, protected time for rest, and structural relief, may help mitigate these additional stressors and support mental health in double-duty carers.

### The impact of age, psychopathology at baseline and of occupation

Finally, in our longitudinal analysis, age between 51 and 60 turned out to be a salutogenic rather than a risk factor for anxiety and depression in the long term. Consistent with this, previous studies have tended to identify younger age as a risk factor for increased psychological distress (63). Finally, inconsistencies in the existing evidence may be due to differences in the age grouping (5, 32).

Above, we identified anxiety/depression at T1 as the most important factor predicting anxiety and depression over the course of the pandemic. Consistent with previous studies, psychopathology at baseline is a good predictor of psychopathology at follow-up (64–66). In addition, our study found that the professional group of MTAs was found to be at higher risk for clinically relevant anxiety and depression, regardless of the caregiving situation. This has been reported previously and may be explained by the increased pressure on MTAs to perform COVID-19 testing, over and above their possibly lower socioeconomic and professional status (33, 34, 67).

### The course of anxiety/depression throughout the pandemic

Our results indicate higher anxiety in ICs compared to the CG throughout the COVID-19 pandemic, and an increase in psychological distress independent of group. This is consistent with increased anxiety and depression already published in physicians (67), but contradicts data showing improved mental health in a cohort of Italian HCWs more than 24 months after the onset of the pandemic (68). The controversial findings may be explained by different primary outcomes (using cut-offs or means) and different time frames. Existing evidence suggests a higher increase in psychological distress in ICs than in non-caregivers (22, 69). For example, in a representative sample during the first COVID-19 lockdown in the United Kingdom (69). In line with this, our data suggest a higher rate of persistent or worsening psychological distress in the IC group when considering clinically relevant symptoms of anxiety and depression (based on cut-off scores) and a time frame of approximately 6 months. However, a definitive conclusion on the course of psychological distress during the phases of the COVID-19 pandemic is not yet possible, as data on anxiety and depression prior to the onset of the pandemic were not available in our present study.

### Strengths and limitations

This study offers valuable insights into the longitudinal mental health trajectories of ICs during the COVID-19 pandemic. Strengths include a large and diverse sample of HCWs across several German university hospitals, as well as a multi-wave design with consistent use of standardized self-report measures across different phases of the pandemic.

Nevertheless, several limitations must be considered. First, the primary outcome measure, the PHQ-4, is a widely used ultrashort screening instrument, but it may lack diagnostic specificity and be susceptible to false-positive responses. Likewise, optimism was measured using a single item, which limits the conceptual and psychometric precision of this construct. A more differentiated assessment, e.g., distinguishing between trait and state optimism using validated scales, would improve theoretical interpretation. Similarly, the constructs “fear of infecting relatives” and “recovery during leisure time” were assessed with self-constructed single items. As these items have not been psychometrically validated, the interpretability of findings is limited and results should be interpreted with caution.

A further limitation lies in the measurement of informal caregiving. Although the binary self-report item used in this study corresponds to conventions in epidemiological population studies and was chosen for feasibility in the context of a large-scale, multi-wave survey, it neglects important caregiving dimensions such as intensity, frequency, duration, relationship to care recipient, and access to external support networks. These factors may have influenced the primary outcomes and a lack of them limits the construct validity of our measure. Future research should therefore incorporate more nuanced, multi-item assessments to adequately capture the complexity of informal caregiving.

Another controversial issue worth mentioning is the definition of informal caregiving as unpaid care for a disabled or seriously ill person (13). This definition excludes care for children or parents who may not be seriously ill, but for whom care responsibilities would certainly increase during a pandemic. Future studies should therefore address other specific risk groups for increased psychological distress, such as those caring for children or parents over 65 years in addition to the seriously ill, outside a pandemic.

The study design also entails methodological constraints. As no information on psychological distress prior to the pandemic was available, we cannot fully disentangle pandemic-specific effects from pre-existing mental health conditions. ICs may have experienced elevated distress levels independent of the pandemic context. Future studies should consider assessing baseline mental health, for example via retrospective self-report of previous diagnoses or treatments.

Although we included longitudinal data, the correlational nature of most variables (e.g., optimism and PHQ-4 assessed at the same time point) precludes causal inference. In addition, optimism was only explored as a moderator in one model. Further studies should examine other salutogenic or resilience-related moderators (e.g., sense of coherence, social support) more systematically and using validated instruments.

With regard to sampling, dropout at follow-up waves, lack of documented response rates, and potential selection effects may limit generalizability. As the sample was primarily composed of German HCWs, cultural and system-specific factors may limit transferability of results to other countries or healthcare systems. Furthermore, although participants generated individual pseudonym codes, we cannot entirely rule out multiple participation at a given time point.

Also, the present analysis is a secondary data analysis from the VOICE study, which was not originally designed to test hypotheses related to informal caregiving. Accordingly, no *a priori* power analysis was conducted for these specific questions. *Post hoc* power analyses suggest that the sample sizes at T3 and T4 were underpowered to detect small effects, and results from these time points should therefore be interpreted with caution. The longitudinal logistic regression analysis included a substantially smaller subsample (*N* = 965; IC: *n* = 153, CG: *n* = 812) which limited the statistical power to detect small effects in longitudinal prediction models. Nevertheless, the impact of IC could be demonstrated in the complete-case analysis, even after controlling for multiple confounders.

In addition to the methodological and measurement limitations outlined above, potential confounding variables should also be considered when interpreting the results. Gender was unevenly distributed between groups and may have contributed to differences in psychological distress, particularly at the onset of the pandemic. Although stratified analyses and multivariate controls mitigate this concern, residual confounding cannot be ruled out. Moreover, unmeasured factors such as pre-pandemic mental health status, occupational stressors (e.g., moral distress, team redeployment), and individual coping resources may have influenced both caregiving status and distress levels. Future research should systematically assess such confounders to better disentangle caregiving-related effects from broader contextual influences.

## Conclusion

HCWs with additional informal caregiving burden (ICs represent a distinct risk group for increased psychological distress during public health crises such as the COVID-19 pandemic). This is particularly true for female ICs, who are disproportionately affected by additional informal caregiving responsibilities, and require targeted support. Mitigation strategies should include a combination of psychological, structural, and employer-based interventions—such as flexible working hours, access to childcare near the workplace, and caregiver leave policies. In addition, psychological support services (e.g., low-threshold counseling, peer groups) and practical resources (e.g., protective equipment, recovery time) are essential to reduce strain and strengthen mental health in this vulnerable group (53).

Beyond the workplace, informal caregiving should receive greater recognition and support at the societal and policy levels to ensure the long-term well-being and work capacity of informally caregiving HCWs—not only during pandemics, but in the face of ongoing demographic and systemic healthcare challenges.

## Data Availability

The raw data supporting the conclusions of this article will be made available by the authors, without undue reservation.
