# Supplementary figures and images for: The impact of informal caregiving on the mental health of health care workers during the COVID-19 pandemic—cross-sectional and longitudinal results from the VOICE study
Source: Front Public Health. 2025 Sep 17;13:1559518. doi: 10.3389/fpubh.2025.1559518 (PMC12486604; doi:10.3389/fpubh.2025.1559518)

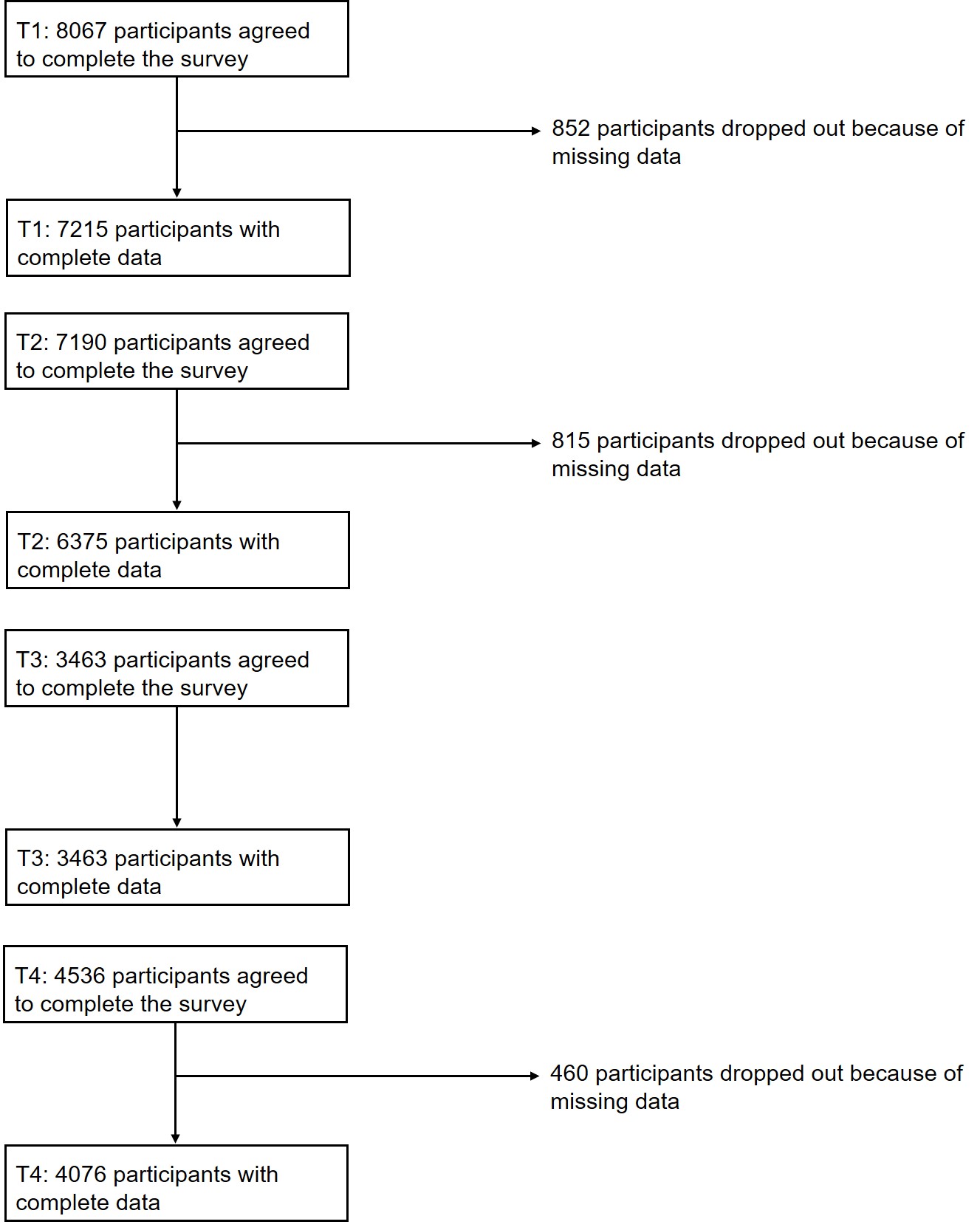

Supplement: Supplementary file 1 [file Image_1.JPEG]

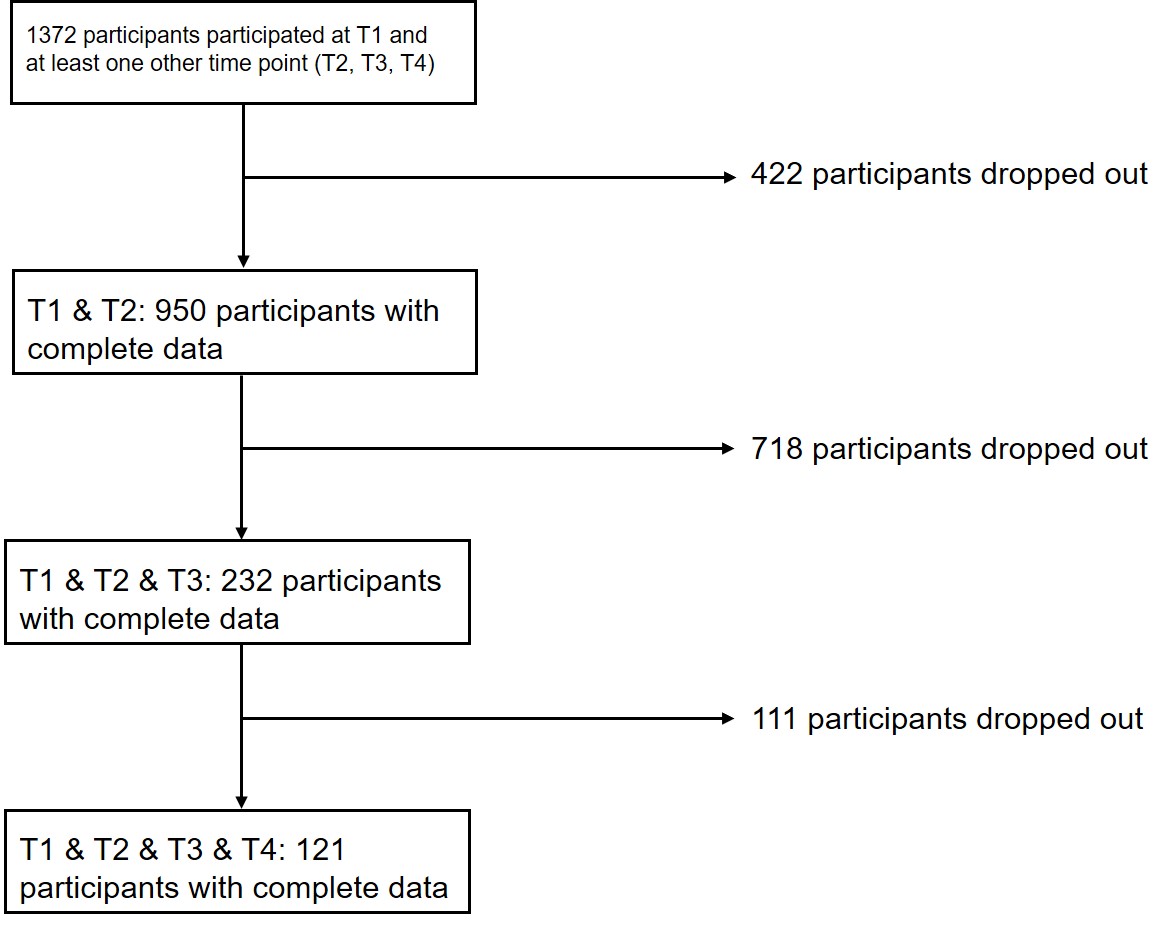

Supplement: Supplementary file 2 [file Image_2.JPEG]

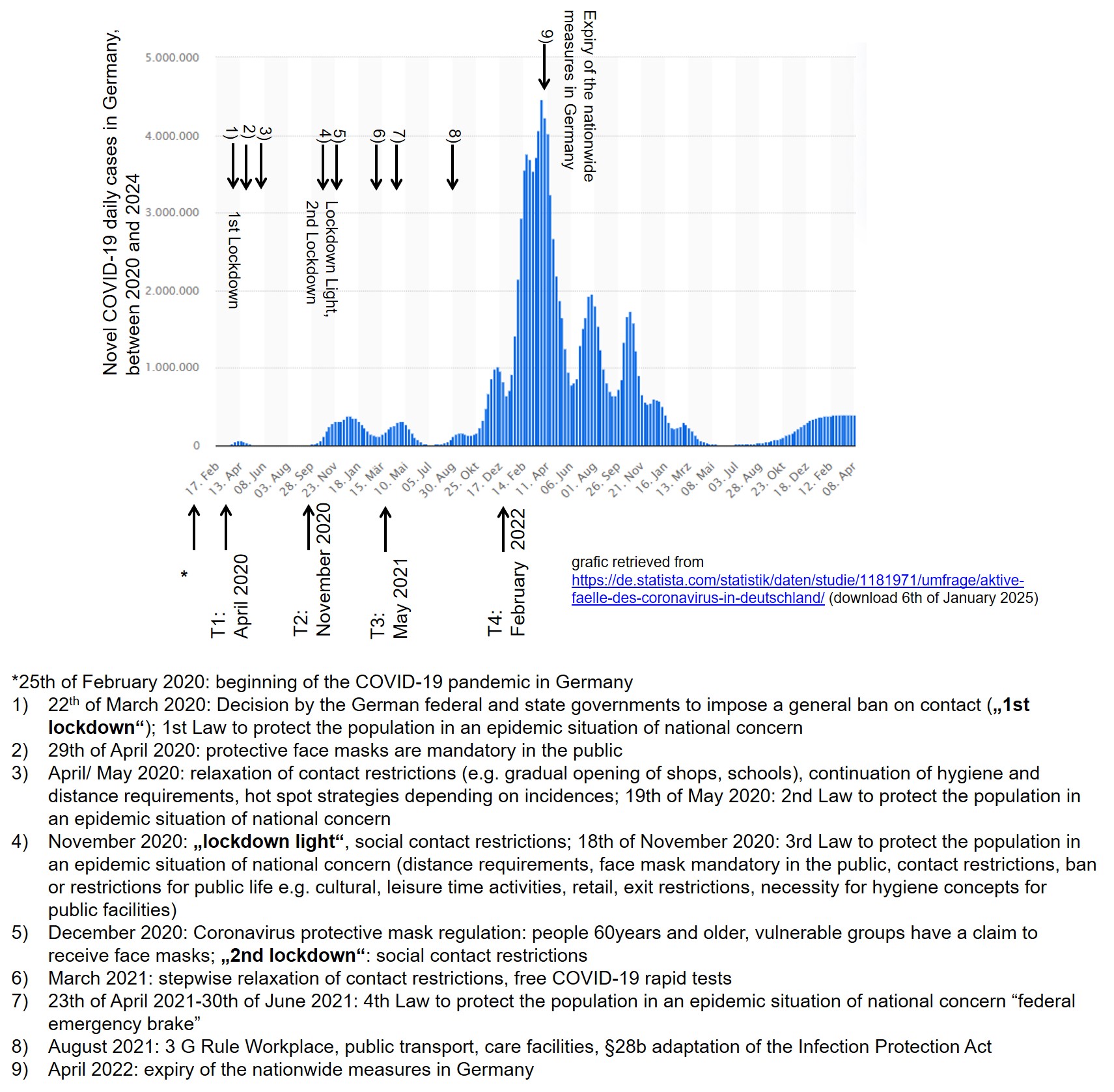

Supplement: Supplementary file 3 [file Image_3.JPEG]
