# Supplementary material for: The impact of informal caregiving on the mental health of health care workers during the COVID-19 pandemic—cross-sectional and longitudinal results from the VOICE study
Source: Front Public Health. 2025 Sep 17;13:1559518. doi: 10.3389/fpubh.2025.1559518 (PMC12486604; doi:10.3389/fpubh.2025.1559518)
Supplement: Supplementary file 4 [file Supplementary_file_1.docx]

**Supplementary Material**

**Figure S1:** Displayed are the types of restrictions and pandemic measures that took place in Germany during the measurement periods (T1, T2, T3, T4) of the VOICE survey. Above, the number of incidence cases of COVID-19 is shown.

**Figure S2_1:** Visualization of the drop-outs for the cross-sectional samples (T1, T2, T3, T4).

**Figure S2_2:** Visualization of the drop-outs for Health Care Workers (HCWs) who participated at least at T1 and one other time point.

**Table S1:** Description of the number and rate (n/ %) of Health Care Workers (HCWs) recruited at different medical workplaces, at different time points (T1: April to July 2020, T2: November 2020 to January 2021, T3: May to July 2021, T4: February to May 2022).

|  | T1 | T2 | T3 | T4 |
| --- | --- | --- | --- | --- |
| University hospital | 3070 (42.6) | 4197 (65.8) | 2548 (73.6) | 2590 (63.5) |
| Other Maximum-care hospital (no university hospital) | 1504 (20.8) | 777 (12.2) | 494 (14.3) | 1067 (26.2) |
| Socio-pediatric center | 1172 (16.2) | 548 (8.6) | -- | -- |
| Doctors’ office | 676 (9.4) | 474 (7.4) | 79 (2.3) | 89 (2.2) |
| Medical care center | 221 (3.1) | 99 (1.6) | 65 (1.9) | 88 (2.2) |
| Other^1^ | 572 (7.9) | 280 (4.4) | 277 (8.0) | 242 (5.9) |

^1^ Other: e.g. occupational health services, nursing homes, outpatient care services, and emergency services

**Table S2:** Dropout analysis comparing Health Care Workers (HCWs) participating at T1 and T2 (= included), and at T1 only (= Drop-outs).

|  | **Included**  n = 950 | **Drop-Outs**  n = 422 | **χ² (p)** |
| --- | --- | --- | --- |
| **Gender, n (%)** |  |  |  |
| female | 200 (78.8) | 331 (78.4) |  |
| male | 749 (21.1) | 91 (21.6) |  |
| divers | 1 (0.1) | 0 (0.0) | .486 (.784) |
| **Age group in years, n (%)** |  |  |  |
| 18-30 | 190 (20.0) | 81 (19.2) |  |
| 31-40 | 219 (23.1) | 91 (21.6) |  |
| 41-50 | 207 (21.8) | 115 (27.3) |  |
| 51-60 | 286 (30.1) | 110 (26.1) |  |
| >60 | 48 (5.1) | 25 (5.9) | 6.165 (.187) |
| **Living alone, n (%)** |  |  |  |
| yes | 220 (23.2) | 95 (22.5) |  |
| no | 730 (76.8) | 327 (77.5) | .069 (.793) |
| **Having children, n (%)** |  |  |  |
| yes | 467 (49.2) | 234 (55.5) |  |
| no | 483 (50.8) | 188 (44.5) | 4.630 (.031)* |
| **Occupation, n (%)** |  |  |  |
| physician | 237 (24.9) | 75 (17.8) |  |
| nurse | 202 (21.3) | 103 (24.4) |  |
| medical-technical assistant | 126 (13.3) | 67 (15.9) |  |
| other^1^ | 385 (40.5) | 177 (41.9) | 9.476 (.024)* |
| **Employment type, n (%)** |  |  |  |
| full-time | 585 (61.6) | 240 (56.9) |  |
| part-time | 365 (38.4) | 182 (43.1) | 2.700 (.100) |
| **Informal caregiving, n (%)** |  |  |  |
| yes | 153 (16.1) | 73 (17.3) |  |
| no | 797 (83.9) | 349 (82.7) | .302 (.582) |
| **Work experience, n (%)^5^** |  |  |  |
| <3 years | 98 (10.3) | 38 (9.0) |  |
| 3-6 years | 107 (11.3) | 48 (11.4) |  |
| >6 years | 594 (62.5) | 268 (63.5) |  |
| Not working in patient care^6^ | 151 (15.9) | 68 (16.1) | .563 (.905) |
| **Direct contact with COVID-19, n (%)^7^** |  |  |  |
| yes | 371 (39.1) | 180 (42.7) |  |
| no | 579 (60.9) | 242 (57.3) | 1.577 (.211) |
| **COVID-19 infection, n (%)** |  |  |  |
| yes | 6 (.6) | 9 (2.1) |  |
| no | 944 (99.4) | 413 (97.9) | 6.089 (.014)* |
| **Primary outcomes,**  **median (interquartile range)**  **mean (SD)** |  |  |  |
| PHQ-4 score | 2.0 (1.0-4.0)  3.0 (2.7) | 2.0 (1.0-4.0)  3.1 (2.8) | 200128.000 (.962) |
| PHQ-2 score | 1.0 (1.0-2.0)  1.6 (1.5) | 1.0 (.0-2.0)  1.6 (1.5) | 196346.000 (.532) |
| GAD-2 score | 1.0 (.0-2.0)  1.4 (1.4) | 1.0 (.0-2.0)  1.5 (1.6) | 196910.500 (.589) |

^1^ other: dentist, paramedic, physiotherapist, psychologist, ergotherapist, speech therapist, pastor, student, scientist, midwife, IT and administration, *p ≤ .05, ** p ≤ .01, ***p ≤ .001

**S3:** Questionnaire of the present study (relevant parts)

| **Patient Health Questionnaire (PHQ)-4:** *Over the last 2 weeks, how often have you been bothered by the following problems?* | | | | |
| --- | --- | --- | --- | --- |
|  | **Not at all** | **Several days** | **More than half the days** | **Nearly every day** |
| 1. Feeling nervous, anxious or on edge. | 0 | 1 | 2 | 3 |
| 1. Not being able to stop or control worrying. | 0 | 1 | 2 | 3 |
| 1. Little interest or pleasure in doing things. | 0 | 1 | 2 | 3 |
| 1. Feeling down, depressed, or hopeless. | 0 | 1 | 2 | 3 |

**Please mark with a cross to what sex you belong.**

| - Female | - Male | - Diverse |
| --- | --- | --- |

**Please mark with a cross to what age group you belong.**

| - 18-30 | - 31-40 | - 41-50 | - 51-60 | - >60 |
| --- | --- | --- | --- | --- |

**Are you living alone, at the moment?**

| - Yes | - No |
| --- | --- |

**Do you care for old, sick or disabled relatives?**

| - Yes, in my own household |
| --- |
| - Yes, but not in my own household |
| - No |

**S0: Questionnaire of the present study (relevant parts), continuation**

**Do you have children?**

| - Yes, in my own household |
| --- |
| - Yes, but not in my own household |
| - No |

**If yes: are you single parent?**

| - Yes | - No |
| --- | --- |

**In which profession are you mainly employed?**

| - Physician/ medical psychotherapist |
| --- |
| - Nurse |
| - Midwife |
| - Medical-Technical assistant |
| - Psychologist/ psychological psychotherapist |
| - Child and adolescent psychotherapist |
| - Pastor |
| - Emergency service |
| - Administrative employee with direct patient contact |
| - Administrative employee without direct patient contact |
| - Information Technology (IT) |
| - Student (medicine) |
| - Student (other academic subject) |
| - Apprentice |
| - Scientific assistant |
| **S0: Questionnaire of the present study (relevant parts), continuation** |
| - Study nurse |
| - Physiotherapist |
| - Occupational therapist |
| - Music therapist |
| - Speech therapist |
| - Social service |
| - Educator |
| - Special education teacher |
| - Others (please write down): |

**Are you working in direct patient care?**

| - Yes | - No |
| --- | --- |

**If yes: How long have you already been working in patient care?**

| - < 3 years | - 3-6 years | - >6 years |
| --- | --- | --- |

**Are working in full- or part-time?**

| - Full-time (100%) |
| --- |
| - Part-time (in percent):_________ |

**During the last two weeks (including today), have you been in direct contact with patients who were infected by SARS-CoV-2 (proved by a corona test), during your work?**

| - Yes | - No |
| --- | --- |

**S0: Questionnaire of the present study (relevant parts), continuation**

**During the last two weeks (including today), have you been in direct contact with material, contaminated by SARS-CoV-2, during your work?**

| - Yes | - No |
| --- | --- |

**Have you been infected with SARS-CoV-2?**

| - Yes | - No | - I do not know. |
| --- | --- | --- |

**To what extent do you agree with the following statements, concerning the working conditions at your workplace, during the last two weeks, including today?**

|  | **I strongly disagree.**  **0** | **I rather do not agree.**  **1** | **I partially agree.**  **2** | **I rather agree.**  **3** | **I strongly agree.**  **4** |
| --- | --- | --- | --- | --- | --- |
| I can recover sufficiently during my free time |  |  |  |  |  |

**Questions concerning potential problems with regard to COVID-19 pandemic.**

*During the last two weeks, including today, ….*

|  | **I strongly disagree.**  **0** | **I rather do not agree.**  **1** | **I partially agree.**  **2** | **I rather agree.**  **3** | **I strongly agree.**  **4** |
| --- | --- | --- | --- | --- | --- |
| I was afraid to become infected. |  |  |  |  |  |
| I was afraid to infect relatives or my family.” |  |  |  |  |  |

**Question regarding general optimism: How optimistic are you in general?**

not at all optimistic 1-----2-----3-----4-----5-----6-----7 very optimistic **Table S4:** Sociodemographic and work-related variables, comparing Health care workers (HCWs) with additional informal care (IC) and without additional informal care (CG = comparison group) responsibility at T2 (between November 2020 to January 2021) (n = 6375)

|  | **IC**  n = 1049 (16.5) | **CG**  n = 5326 (83.5) | **χ² (p)** |
| --- | --- | --- | --- |
| **Gender, n (%)** |  |  |  |
| Female | 801 (76.4) | 3870 (72.7) |  |
| Male | 242 (23.1) | 1441 (27.1) |  |
| Divers | 6 (0.6) | 15 (0.3) | 9.152 (.010)* |
| **Age group in years, n (%)** |  |  |  |
| 18-30 | 126 (12.0) | 1320 (24.8) |  |
| 31-40 | 138 (13.2) | 1400 (26.3) |  |
| 41-50 | 243 (23.2) | 1169 (21.9) |  |
| 51-60 | 443 (42.2) | 1112 (20.9) |  |
| >60 | 99 (9.5) | 325 (6.1) | 306.241 (<.001)*** |
| **Living alone, n (%)** |  |  |  |
| Yes | 244 (23.3) | 1281 (24.1) |  |
| No | 805 (76.7) | 4045 (75.9) | 0.302 (.583) |
| **Having children, n (%)** |  |  |  |
| Yes | 646 (61.6) | 2588 (48.6) |  |
| No | 403 (38.4) | 2738 (51.4) | 59.171 (<.001)*** |
| **Occupation, n (%)** |  |  |  |
| physician | 240 (22.9) | 1330 (25.0) |  |
| Nurse | 289 (27.6) | 1167 (21.9) |  |
| medical-technical assistant | 129 (12.3) | 691 (13.0) |  |
| other^1^ | 391 (37.3) | 2138 (40.1) | 15.889 (.001)** |
| **Employment type, n (%)** |  |  |  |
| full-time | 644 (61.4) | 3434 (64.5) |  |
| part-time | 405 (38.6) | 1892 (35.5) | 3.617 (.057) |
| **Work experience, n (%)^5^** |  |  |  |
| <3 years | 63 (6.0) | 624 (11.7) |  |
| 3-6 years | 79 (7.5) | 742 (13.9) |  |
| >6 years | 721 (68.7) | 2939 (55.2) |  |
| Not working in patient care^6^ | 186 (17.7) | 1021 (19.2) | 83.383 (<.001)*** |
| **Direct contact with COVID-19, n (%)^7^** |  |  |  |
| Yes | 552 (52.6) | 2517 (47.3) |  |
| No | 497 (47.4) | 2809 (52.7) | 10.096 (.001)** |
| **COVID-19 infection, n (%)** |  |  |  |
| Yes | 29 (2.8) | 125 (2.3) |  |
| No | 1020 (97.2) | 5201 (97.7) | .648 (.421) |

^1^ other: dentist, paramedic, physiotherapist, psychologist, ergotherapist, speech therapist, pastor, student, scientist, midwife, IT and administration, CG = Comparison Group, IG = Informal Caregivers, *p ≤ .05, ** p ≤ .01, ***p ≤ .001

**Table S5:** Sociodemographic and work-related variables, comparing Health care workers (HCWs) with additional informal care (IC) and without additional informal care (CG = comparison group) responsibility at T3 (between May to July 2021) (n = 3463)

|  | **IC**  n = 542 (15.7) | **CG**  n = 2921 (84.3) | **χ² (p)** |
| --- | --- | --- | --- |
| **Gender, n (%)** |  |  |  |
| Female | 427 (78.8) | 2192 (75.0) |  |
| Male | 115 (21.2) | 722 (24.7) |  |
| Divers | 0 (0.0) | 7 (0.2) | 4.461 (.107) |
| **Age group in years, n (%)** |  |  |  |
| 18-30 | 53 (9.8) | 659 (22.6) |  |
| 31-40 | 65 (12.0) | 726 (24.9) |  |
| 41-50 | 109 (20.1) | 645 (22.1) |  |
| 51-60 | 254 (46.9) | 688 (23.6) |  |
| >60 | 61 (11.3) | 203 (6.9) | 172.688 (<.001)*** |
| **Living alone, n (%)** |  |  |  |
| Yes | 119 (22.0) | 705 (24.1) |  |
| No | 423 (78.0) | 2216 (75.9) | 1.198 (.274) |
| **Having children, n (%)** |  |  |  |
| Yes | 325 (60.0) | 1423 (48.7) |  |
| No | 217 (40.0) | 1498 (51.3) | 23.134 (<.001)*** |
| **Occupation, n (%)** |  |  |  |
| Physician | 67 (17.5) | 496 (17.0) |  |
| Nurse | 128 (33.5) | 627 (21.5) |  |
| medical-technical assistant | 41 (10.7) | 304 (10.4) |  |
| other^1^ | 306 (56.5) | 1494 (51.1) | 13.159 (.004)** |
| **Employment type, n (%)** |  |  |  |
| full-time | 326 (60.1) | 1820 (62.3) |  |
| part-time | 216 (39.9) | 1101 (37.7) | .905 (.341) |
| **Work experience, n (%)^5^** |  |  |  |
| <3 years | 24 (6.5) | 206 (10.8) |  |
| 3-6 years | 24 (6.5) | 319 (16.7) |  |
| >6 years | 319 (86.9) | 1380 (72.4) |  |
| Not working in patient care^6^ |  |  | 35.412 (<.001)*** |
| **Direct contact with COVID-19, n (%)^7^** |  |  |  |
| Yes | 132 (24.4) | 637 (21.8) |  |
| No | 410 (75.6) | 2284 (78.2) | 1.716 (.190) |
| **COVID-19 infection, n (%)** |  |  |  |
| Yes | 40 (7.4) | 179 (6.1) |  |
| No | 502 (92.6) | 2742 (93.9) | 1.210 (.271) |

^1^ other: dentist, paramedic, physiotherapist, psychologist, ergotherapist, speech therapist, pastor, student, scientist, midwife, IT and administration, CG = Comparison Group, IG = Informal Caregivers, *p ≤ .05, ** p ≤ .01, ***p ≤ .001

**Table S6:** Sociodemographic and work-related variables, comparing Health care workers (HCWs) with additional informal care (IC) and without additional informal care (CG = comparison group) responsibility at T4 (between February to May 2022) (n = 4076)

|  | **IC**  n = 659 (16.2) | **CG**  3417 (83.8) | **χ² (p)** |
| --- | --- | --- | --- |
| **Gender, n (%)** |  |  |  |
| Female | 513 (77.8) | 2576 (75.4) |  |
| Male | 143 (21.7) | 835 (24.4) |  |
| Divers | 3 (0.5) | 6 (0.2) | 4.122 (.127) |
| **Age group in years, n (%)** |  |  |  |
| 18-30 | 76 (11.5) | 770 (22.5) |  |
| 31-40 | 98 (14.9) | 871 (25.5) |  |
| 41-50 | 146 (22.2) | 800 (23.4) |  |
| 51-60 | 279 (42.3) | 772 (22.6) |  |
| >60 | 60 (9.1) | 204 (6.0) | 150.700 (<.001)*** |
| **Living alone, n (%)** |  |  |  |
| Yes | 144 (21.9) | 836 (24.5) |  |
| No | 515 (78.1) | 2581 (75.5) | 2.068 (.150) |
| **Having children, n (%)** |  |  |  |
| Yes | 380 (57.7) | 1753 (51.3) |  |
| No | 279 (42.3) | 1664 (48.7) | 8.960 (.003)** |
| **Occupation, n (%)** |  |  |  |
| physician | 69 (10.5) | 589 (17.2) |  |
| Nurse | 186 (28.2) | 824 (24.1) |  |
| medical-technical assistant | 81 (12.3) | 443 (13.0) |  |
| other^1^ | 323 (49.0) | 1561 (45.7) | 20.957 (<.001)*** |
| **Employment type, n (%)** |  |  |  |
| full-time | 397 (60.2) | 2129 (62.3) |  |
| part-time | 262 (39.8) | 1288 (37.7) | .998 (.318) |
| **Work experience, n (%)^5^** |  |  |  |
| <3 years | 25 (5.6) | 284 (11.9) |  |
| 3-6 years | 49 (11.0) | 364 (15.3) |  |
| >6 years | 370 (83.3) | 1731 (72.8) |  |
| Not working in patient care^6^ |  |  | 23.868 (<.001)*** |
| **Direct contact with COVID-19, n (%)^7^** |  |  |  |
| Yes | 355 (53.9) | 1725 (50.5) |  |
| No | 304 (46.1) | 1692 (49.5) | 2.536 (.111) |
| **COVID-19 infection, n (%)** |  |  |  |
| Yes | 164 (24.9) | 960 (28.1) |  |
| No | 495 (75.1) | 2457 (71.9) | 2.848 (.091) |

^1^ other: dentist, paramedic, physiotherapist, psychologist, ergotherapist, speech therapist, pastor, student, scientist, midwife, IT and administration, CG = Comparison Group, IG = Informal Caregivers, *p ≤ .05, ** p ≤ .01, ***p ≤ .001

**Table S7:** Intensity of anxiety and depression (PHQ-4), depression (PHQ-2) and anxiety (GAD-2) at T1, T2, T3, and T4. Cross-sectional analyses with data after multiple imputation. The number of imputed data for PHQ were n = 852 (10.6%) at T1, n = 815 (11.3%) at T2, at T3 no data were imputed, n = 460 (10.1%) at T4.

|  | **IC, mean (SD)** | **CG, mean (SD)** | **U (p)** | **Coefficient of determination R²** |
| --- | --- | --- | --- | --- |
| *PHQ-4* | | | |  |
| T1^1^ | 3.47 (2.76) | 3.22 (2.72) | 4570793.500 (<.001***) .003**# | .002 |
| T2^2^ | 4.06 (2.89) | 3.70 (2.87) | 3227874.000 (<.001***) .003**# | .003 |
| T3^3^ | 3.62 (2.74) | 3.58 (2.86) | 771246.000 (.338) .676# | .000 |
| T4^4^ | 4.04 (2.93) | 3.83 (2.87) | 1310883.500 (.051) .102# | .001 |
| *PHQ-2* | | | |  |
| T1^1^ | 1.72 (1.46) | 1.60 (1.45) | 4599768.500 (.001***) .003**# | .001 |
| T2^2^ | 2.08 (1.53) | 1.95 (1.54) | 3331476.000 (.004**) .004**# | .001 |
| T3^3^ | 1.92 (1.50) | 1.91 (1.53) | 784626.000 (.738) .738# | .000 |
| T4^4^ | 2.08 (1.56) | 2.00 (1.52) | 1337531.500 (.252) .252# | .000 |
| *GAD-2* | | | |  |
| T1^1^ | 1.72 (1.55) | 1.51 (1.50) | 4428662.000 (<.001***) .003**# | .004 |
| T2^2^ | 1.97 (1.61) | 1.74 (1.59) | 3191784.500 (<.001***) .003**# | .004 |
| T3^3^ | 1.70 (1.50) | 1.67 (1.59) | 768603.000 (.270) .810# | .000 |
| T4^4^ | 1.92 (1.62) | 1.75 (1.56) | 1294067.000 (.012*) .036*# | .001 |

^1^ IC: n = 1474, CG: n = 6587; ^2^ IC: n = 1167, CG: n = 6022; ^3^ IC: n = 542, CG: n = 2921, Note that at measurement point T3, only complete cases without missing data were included in the dataset. This decision was made in accordance with the analytical strategy of the time, which prioritized including fully complete data. However, this approach was later abandoned as it proved impractical for the other measurement points. Consequently, there are no missing values recorded for T3 because all incomplete cases were excluded from the dataset.; ^4^ IC: n = 720, CG: n = 3815; *p≤.05; **p≤.01; #Bonferroni-Holm-corrected p-values; CG = Comparison Group; GAD = Generalized Anxiety Disorder; IC = Informal Caregivers; PHQ = Patient Health Questionnaire

**Table S8:** Intensity of anxiety and depression (PHQ-4), depression (PHQ-2) and anxiety (GAD-2) at T1, T2, T3, and T4. Cross-sectional analyses with data after multiple imputation, including only men. The number of imputed data for PHQ were n = 220 (11.5%) at T1, n = 228 (11.9%) at T2, at T3 no data were imputed, n = 107 (9.9%) at T4.

|  | **IC, mean (SD)** | **CG, mean (SD)** | **U (p)** | **Coefficient of determination R²** |
| --- | --- | --- | --- | --- |
| *PHQ-4* | | | |  |
| T1^1^ | 3.00 (2.64) | 2.89 (2.67) | 216868.500 (.404) .808# | .000 |
| T2^2^ | 4.05 (3.10) | 3.42 (2.98) | 194430.000 (<.001***) .003**# | .007 |
| T3^3^ | 3.27 (2.73) | 3.28 (2.82) | 40970.500 (.820) 1.000# | .000 |
| T4^4^ | 3.79 (3.09) | 3.49 (2.83) | 67850.500 (.333) .666# | .001 |
| *PHQ-2* | | | |  |
| T1^1^ | 1.54 (1.43) | 1.51 (1.45) | 220149.000 (.651) .651**# | .000 |
| T2^2^ | 2.18 (1.65) | 1.90 (1.59) | 200965.500 (.005**) .004**# | .004 |
| T3^3^ | 1.83 (1.49) | 1.85 (1.53) | 41304.500 (.928) .928# | .000 |
| T4^4^ | 2.14 (1.73) | 2.02 (1.59) | 69266.500 (.563) .563# | .000 |
| *GAD-2* | | | |  |
| T1^1^ | 1.36 (1.43) | 1.28 (1.43) | 216366.500 (.356) 1.000# | .000 |
| T2^2^ | 1.87 (1.70) | 1.53 (1.61) | 195559.000 (<.001***) .003**# | .006 |
| T3^3^ | 1.44 (1.52) | 1.43 (1.51) | 40961.000 (.812) 1.000# | .000 |
| T4^4^ | 1.81 (1.69) | 1.60 (1.54) | 66620.000 (.180) .540# | .002 |

^1^ IC: n = 272, CG: n = 1646; ^2^ IC: n = 274, CG: n = 1636; ^3^ IC: n = 115, CG: n = 722, Note that at measurement point T3, only complete cases without missing data were included in the dataset. This decision was made in accordance with the analytical strategy of the time, which prioritized including fully complete data. However, this approach was later abandoned as it proved impractical for the other measurement points. Consequently, there are no missing values recorded for T3 because all incomplete cases were excluded from the dataset.; ^4^ IC: n = 153, CG: n = 932; *p≤.05; **p≤.01; #Bonferroni-Holm-corrected p-values; CG = Comparison Group; GAD = Generalized Anxiety Disorder; IC = Informal Caregivers; PHQ = Patient Health Questionnaire

**Table S9:** Intensity of anxiety and depression (PHQ-4), depression (PHQ-2) and anxiety (GAD-2) at T1, T2, T3, and T4. Cross-sectional analyses with data after multiple imputation, including only women. The number of imputed data for PHQ were n = 616 (10.1%) at T1, n = 583 (11.1%) at T2, at T3 no data were imputed, n = 347 (10.1%) at T4.

|  | **IC, mean (SD)** | **CG, mean (SD)** | **U (p)** | **Coefficient of determination R²** |
| --- | --- | --- | --- | --- |
| *PHQ-4* | | | |  |
| T1^1^ | 3.58 (2.77) | 3.32 (2.72) | 2757702.500 (.002**) .004**# | .002 |
| T2^2^ | 4.06 (2.89) | 3.70 (2.87) | 1813023.500 (.004**) .008**# | .002 |
| T3^3^ | 3.72 (2.74) | 3.68 (2.87) | 462986.500 (.410) .820# | .000 |
| T4^4^ | 4.09 (2.89) | 3.93 (2.86) | 776925.000 (.155) .310# | .001 |
| *PHQ-2* | | | |  |
| T1^1^ | 1.75 (1.46) | 1.62 (1.45) | 2767813.500 (.002**) .004**# | .002 |
| T2^2^ | 2.08 (1.53) | 1.95 (1.54) | 1862751.500 (.094) .094# | .001 |
| T3^3^ | 1.94 (1.51) | 1.93 (1.53) | 454932.000 (.719) .719# | .000 |
| T4^4^ | 2.05 (1.49) | 1.99 (1.49) | 788411.500 (.365) .365# | .000 |
| *GAD-2* | | | |  |
| T1^1^ | 1.81 (1.56) | 1.58 (1.52) | 2657041.000 (<.001***) .003**# | .004 |
| T2^2^ | 1.97 (1.61) | 1.74 (1.59) | 1785505.000 (<.001***) .003**# | .002 |
| T3^3^ | 1.77 (1.49) | 1.76 (1.61) | 454932.000 (.349) 1.000# | .000 |
| T4^4^ | 1.94 (1.61) | 1.80 (1.56) | 767213.000 (.056) .168# | .001 |

^1^ IC: n = 1190, CG: n = 4922; ^2^ IC: n = 883, CG: n = 4371; ^3^ IC: n = 427, CG: n = 2192, Note that at measurement point T3, only complete cases without missing data were included in the dataset. This decision was made in accordance with the analytical strategy of the time, which prioritized including fully complete data. However, this approach was later abandoned as it proved impractical for the other measurement points. Consequently, there are no missing values recorded for T3 because all incomplete cases were excluded from the dataset.; ^4^ IC: n = 562, CG: n = 2873; *p≤.05; **p≤.01; #Bonferroni-Holm-corrected p-values; CG = Comparison Group; GAD = Generalized Anxiety Disorder; IC = Informal Caregivers; PHQ = Patient Health Questionnaire

**Table S10:** The influencing variables of clinically relevant anxiety and depression (PHQ-4 ≥ 6) were examined in a longitudinal sample including all participants with complete data at both T1 and T2, as well as those with missing data on the primary outcome (data missing at T1 and/or T2). The sample size was N = 1406, including IC (n = 229) and CG (n = 1177).

|  | **Regression coefficient B** | **SE** | **Wald** | **p** | **OR (95%CI)** |
| --- | --- | --- | --- | --- | --- |
| *Informal caregiving* | | | | | |
| Yes | .482 | .197 | 5.972 | **.015*** | 1.620 (1.100, 2.385) |
| *Gender* | | | | | |
| Female | -.462 | .177 | 6.789 | **.009**** | .630 (.445, .892) |
| Gender x Informal Caregiving | -.841 | .507 | 2.757 | .097 | .431 (.160, 1.164) |
| *Age (years)* | | | | | |
| 31-40 | .144 | .232 | .386 | .535 | 1.155 (.733, 1.818) |
| 41-50 | -.737 | .273 | 7.301 | **.007**** | .479 (.280, .817) |
| 51-60 | -.396 | .261 | 2.297 | .130 | .673 (.403, 1.123) |
| >60 | .230 | .352 | .425 | .514 | 1.258 (.631, 2.509) |
| *Living alone* | | | | | |
| Yes | -.144 | .169 | .730 | .393 | .866 (.622, 1.205) |
| *Children* | | | | | |
| Yes | .046 | .172 | .072 | .789 | 1.047 (.747, 1.467) |
| Occupation |  |  |  |  |  |
| Nursing | .494 | .205 | 5.799 | **.016*** | 1.639 (1.096, 2.449) |
| MTA | .174 | .238 | .535 | .465 | 1.190 (.747, 1.895) |
| Other | -.099 | .199 | .248 | .618 | .906 (.613, 1.337) |
| *Full-time* | | | | | |
| Yes | .041 | .155 | .071 | .790 | 1.042 (.769, 1.411) |
| *Work experience (years)* | | | | | |
| 3-6 | -.138 | .286 | .233 | .629 | .871 (.497, 1.527) |
| >6 | -.199 | .276 | .520 | .471 | .820 (.478, 1.407) |
| Not working with patients | .296 | .296 | 1.001 | .317 | 1.344 (.753, 2.399) |
| *Contact with COVID-19* | | | | | |
| Yes | .288 | .142 | 4.093 | **.043*** | 1.333 (1.009, 1.762) |
| *PHQ-4 ≥ 6 at T1* | 1.385 | .160 | 79.944 | **<.001***** | 3.994 (2.919, 5.465) |
| Nagelkerkes R² = .150, -2 Loglikelihood = 1368.774, ꭓ² = 3.721, df = 8, p = .881 | | | | | |

CG = Comparison Group, GAD = Generalized Anxiety Disorder, IG = Informal Caregivers, MTA = medical technical assistant, PHQ = Patient Health Questionnaire

**Table S11:** The secondary outcome variables after multiple imputation: fear of becoming infected, the fear of infecting relatives or family members, and the ability to recover during leisure time were compared between informal caregivers (ICs) and a comparison group of healthcare workers without caregiving responsibilities (CG). Each item was rated on a 5-point Likert scale ranging from 1 ("strongly disagree") to 5 ("strongly agree"). Cross-sectional analyses were performed on data after multiple imputation. 1554 (19.3%)/ 2044 (28.4%)/ 167 (3.7%) missing data were imputed for “recovery during leisure time”, 497 (6.2%)/ 510 (7.1%)/ 881 (19.4%) “for fear of becoming infected” and “fear of infecting relatives”, at T1/ T2/ T4.

|  | **IC, mean (SD)** | **CG, mean (SD)** | **U (p)** | **Coefficient of determination R²** |
| --- | --- | --- | --- | --- |
| *Fear of becoming infected* | | | |  |
| T1^1^ | 2.86 (1.27) | 2.60 (1.22) | 4287922.500 (<.001***) .003**# | .006 |
| T2^2^ | 3.21 (1.26) | 3.06 (1.26) | 21423921.500 (<.001***) .003**# | .002 |
| T3^3^ | 2.24 (1.17) | 2.12 (1.12) | 747583.500 (.031*) .062# | .001 |
| T4^4^ | 2.92 (1.35) | 2.85 (1.37) | 1331106.000 (.038*) .180# | .000 |
| *Fear of infecting relatives or family* | | | |  |
| T1^1^ | 3.63 (1.28) | 3.20 (1.35) | 3981341.500 (<.001***) .003**# | .015 |
| T2^2^ | 3.86 (1.19) | 3.53 (1.30) | 21148138.000 (<.001***) .003**# | .009 |
| T3^5^ | 2.83 (1.41) | 2.59 (1.35) | 711333.000 (<.001***) .003**# | .004 |
| T4^4^ | 3.49 (1.35) | 3.19 (1.39) | 1204320.000 (<.001***) .003**# | .006 |
| *Recovery during leisure time* | | | |  |
| T1^1^ | 3.00 (1.27) | 3.07 (1.28) | 4693940.000 (.042*) .042*# | .001 |
| T2^2^ | 2.56 (1.21) | 2.71 (1.20) | 3935930.500 (<.001***) .003**# | .002 |
| T3^3^ | 2.75 (1.23) | 2.81 (1.22) | 767863.500 (.254) .254# | .000 |
| T4^4^ | 2.63 (1.15) | 2.75 (1.18) | 1289447.000 (.007**) .014*# | .002 |

^1^ IC: n = 1474, CG: n = 6587; ^2^ IC: n =1167, CG: n = 6022; ^3^ IC: n = 542, CG: n = 2921; ^4^ IC: n = 720, CG: n = 3815; ^5^ IC: n = 539, CG: n = 2911; Note that at measurement point T3, only complete cases without missing data were included in the dataset. This decision was made in accordance with the analytical strategy of the time, which prioritized including fully complete data. However, this approach was later abandoned as it proved impractical for the other measurement points. Consequently, there are no missing values recorded for T3 because all incomplete cases were excluded from the dataset; **p≤.01; ***p≤.001; #Bonferroni-Holm-corrected p-values; CG = Comparison Group, GAD = Generalized Anxiety Disorder, IG = Informal Caregivers, PHQ = Patient Health Questionnaire

**Table S12A:** Conditional effects of the focal predictor (optimism: x) at values of the moderator (fear of infecting relatives: w) for the outcome anxiety/ depression (measure: PHQ-4), complete data (n = 152).

LLCI : Lower Limit Confidence Interval, ULCI: Upper Limit Confidence Interval; PHQ: Patient Health Questionnaire; se = standard error

**Table S12B:** Conditional effect of the focal predictor (optimism: x) at values of the moderator (fear of infecting relatives: w) for the outcome anxiety/ depression (measure: PHQ-4), complete data (n = 152).

Moderator value(s) defining Johnson-Neyman significance region(s):

Value % below % above

,6257 67,1053 32,8947

LLCI : Lower Limit Confidence Interval, ULCI: Upper Limit Confidence Interval; PHQ: Patient Health Questionnaire; se = standard error

**Table S13A:** Conditional effects of the focal predictor (optimism: x) at values of the moderator (fear of infecting relatives: w) for the outcome depression (measure: PHQ-2), completed data (n = 152).

LLCI : Lower Limit Confidence Interval, ULCI: Upper Limit Confidence Interval; PHQ: Patient Health Questionnaire; se = standard error

**Table S13B:** Conditional effect of the focal predictor (optimism: x) at values of the moderator (fear of infecting relatives: w) for the outcome depression (measure: PHQ-2).

Moderator value(s) defining Johnson-Neyman significance region(s):

Value % below % above

,8333 67,1053 32,8947

LLCI : Lower Limit Confidence Interval, ULCI: Upper Limit Confidence Interval; PHQ: Patient Health Questionnaire; se = standard error

**Table S14A:** Conditional effects of the focal predictor (optimism: x) at values of the moderator (fear of infecting relatives: w) for the outcome anxiety (measure: GAD-2), complete data (n = 152).

LLCI : Lower Limit Confidence Interval, ULCI: Upper Limit Confidence Interval; PHQ: Patient Health Questionnaire; se = standard error

**Table S14B:** Conditional effect of the focal predictor (optimism: x) at values of the moderator (fear of infecting relatives: w) for the outcome anxiety (measure: GAD-2), complete data (n = 152).

Moderator value(s) defining Johnson-Neyman significance region(s):

Value % below % above

,8333 67,1053 32,8947

LLCI : Lower Limit Confidence Interval, ULCI: Upper Limit Confidence Interval; PHQ: Patient Health Questionnaire; se = standard error

**Table S15:** An additional moderation analysis was conducted that included participants with complete primary outcome data (at both T1 and T2) as well as participants with missing data at either T1 or T2. The following analysis examined the salutogenic effect of optimism at T1 on anxiety and depression (PHQ-4), depression (PHQ-2), and anxiety (GAD-2) at T2. This effect was moderated by fear of infecting family or relatives at T1. The analysis was conducted on the group of informal caregivers only (IC, n = 224).

| **Regressors** | **Outcome** | **Beta (95% CI)** | **t** | **P** |
| --- | --- | --- | --- | --- |
| General Optimism at T1 | GAD-2 | -.154 (-.307, -.001) | -1.986 | .048* |
|  | PHQ-2 | -.256  (-.415, -.096) | -3.154 | .002** |
|  | PHQ-4 | -.492  (-.789, -.194) | -3.257 | .001** |
| Fear of infecting relatives at T1 (moderator) | GAD-2 | .325 (.160, .489) | 3.892 | <.001*** |
|  | PHQ-2 | .119  (-.053, .291) | 1.365 | .174 |
|  | PHQ-4 | .281  (-.039, .602) | 1.732 | .085 |
| Interaction fear x General Optimism | GAD-2 | .133 (.004, .262) | 2.034 | .043* |
|  | PHQ-2 | .056  (-.079, .190) | .811 | .418 |
|  | PHQ-4 | .304  (.053, .555) | 2.386 | .018* |

*p≤.05, **p≤.01, ***p≤.001, CI = Confidence Interval
